# Multi‐omic data integration and exploiting metabolic models using systems biology approach increase precision in subtyping and early diagnosis of cancer

**DOI:** 10.1002/qub2.70012

**Published:** 2025-08-05

**Authors:** Ezgi Tanıl, Emrah Nikerel

**Affiliations:** ^1^ Department of Genetics and Bioengineering Yeditepe University Istanbul Türkiye

**Keywords:** genome scale metabolic model, lung cancer, machine learning, marker pathway enrichment, multi omics data classification, pancreatic cancer

## Abstract

Cancer is a complex and heterogeneous disease characterized by various genetic and epigenetic alterations. Early diagnosis, accurate subtyping, and staging are essential for effective, personalized treatment and improved survival rates. Traditional diagnostic methods, such as biopsies, are invasive and carry operational risks that hinder repeated use, underscoring the need for noninvasive and personalized alternatives. In response, this study integrates transcriptomic data into human genome‐scale metabolic models (GSMMs) to derive patient‐specific flux distributions, which are then combined with genomic, proteomic, and fluxomic (JX) data to develop a robust multi‐omic classifier for lung cancer subtyping and early diagnosis. The JX classifier is further enhanced by analyzing heterogeneous datasets from RNA sequencing and microarray analyses derived from both tissue samples and cell culture experiments, thereby enabling the identification of key marker features and enriched pathways such as lipid metabolism and energy production. This integrated approach not only demonstrates high performance in distinguishing lung cancer subtypes and early‐stage disease but also proves robust when applied to limited pancreatic cancer data. By linking genotype to phenotype, GSMM‐driven flux analysis overcomes challenges related to metabolome data scarcity and platform variability by proposing marker processes and reactions for further investigation, ultimately facilitating noninvasive diagnostics and the identification of actionable biomarkers for targeted therapeutic intervention. These findings offer significant promise for streamlining clinical workflows and enabling personalized therapeutic strategies, and they highlight the potential of our versatile workflow for unveiling novel biomarker landscapes in less studied diseases.

## 1 INTRODUCTION

Cancer is a complex disease and the second most common cause of death globally. Lung cancer, the leading cause of cancer‐related deaths, accounts for 12% of total new cancer diagnoses and 20% of cancer‐related deaths [1]. Despite advancements in treatment, the 5‐year survival rate remains approximately 26%, primarily due to challenges in distinguishing it from other pulmonary diseases. Early diagnosis significantly improves this rate to 85% [2]. Pancreatic cancer (PC), although less prevalent at 3.3% of new diagnoses, ranks as the third deadliest cancer, contributing to 8.5% of cancer‐related deaths [3]. Its high mortality rate stems from asymptomatic early stages and rapid progression, compounded by the lack of effective screening tools [4]. These statistics underscore the need for accurate early detection and personalized treatment to improve patient survival rates.

Current methods for cancer detection and monitoring, such as biopsies, imaging‐guided procedures, and endoscopic approaches, are critical yet invasive, with operational risks that limit their repeated use. Noninvasive methods, such as liquid biopsy, offer safer, more practical alternatives for frequent monitoring and population‐wide screening. Therefore, finding markers directly measurable by liquid biopsy and are specific to the disease/subtype/stage/treatment response is essential [5, 6]. By detecting biomarkers, such as circulating tumor DNA, circulating tumor cells, exosomes, or metabolites, liquid biopsies enable real‐time assessment of disease type, treatment response, and potential recurrence [7, 8, 9]. However, identifying disease‐specific biomarkers for subtyping and staging remains a challenge, often hindered by the complexity of tumor heterogeneity, sample variability, and the low abundance of certain biomarkers [10, 11, 12, 13, 14, 15, 16, 17, 18].

Metabolite biomarkers, in particular, hold unique advantages. Sensitive to metabolic changes, they provide real‐time insights into the body’s state, making them suitable for dynamic monitoring. Their cost‐effectiveness and scalability allow for repeated testing and population‐wide applications [5, 7, 8, 19]. Despite their potential, the identification of reliable metabolite biomarkers faces several hurdles, including the scarcity of metabolome data, dynamic variability, and technical challenges in measurement methods. A solution to this challenge involves identifying marker pathways and reactions for systems‐level analyses of metabolites, rather than relying solely on data from complex profiling studies. To investigate the affected pathways and interaction modules, methods based on metabolite mapping, statistical analysis, and enrichment have been developed. However, most of these methods depend on the analysis of limited metabolome data and the relative changes in metabolite expressions, without providing information on the absolute expression values. Therefore, it is crucial to consider data from various omic levels for a comprehensive understanding [14, 20, 21].

Advances in multi‐omic data integration, driven by statistical, machine learning (ML), and network‐based methods, have enabled comprehensive analyses of complex diseases such as cancer [22, 23]. While these methods have predominantly focused on genomic (GX) and transcriptomic (TX) markers, metabolomic data remains underutilized [10, 13, 14, 18, 24, 25, 26, 27]. High‐throughput profiling and experimental approaches face challenges such as biases, variable data quality, and reduced reproducibility, particularly in metabolome‐based classifiers [20, 21]. To address these issues, genome‐scale metabolic models (GSMMs) offer an alternative by integrating diverse omic data to identify pathways and reactions associated with specific disease states [22, 28, 29].

Tumor cells exhibit altered metabolism, the most notable example being the Warburg effect [30]. These alterations occur across GX, TX, fluxomic (JX), and metabolomic levels, necessitating systems biology approaches for meaningful analysis. GSMMs represent a comprehensive network of biochemical reactions that occur within an organism, based on its genome and they are used to simulate intracellular flux distributions under specific conditions using methods such as flux balance analysis (FBA) [31]. These models optimize objective functions (e.g., growth rate) and refine solutions through techniques such as flux variability analysis [32] or parsimonious FBA [33]. Integrating omic data, including TX and JX layers, further constrains GSMMs, enabling the identification of reporter reactions linked to cancer subtypes and stages.

The integration of JX data, such as ^13^C flux measurements, provides direct insights into intracellular flux distributions [34]. Alternatively, TX data can be used to constrain GSMMs, either assuming gene expression levels correlate with enzyme activity such as E‐flux [35] and Lee‐12 [36], or using context‐specific modeling techniques such as gene inactivity moderated by metabolism and expression (GIMME) [37], The integrative network inference for tissues (INIT) [38], and iMAT [39] refine GSMM simulations by narrowing the solution space and removing unused pathways. The integration of high‐throughput cancer‐related omic data with GSMMs has gained significance in studying complex diseases. This approach enables the determination of patient‐specific fluxome profiles and identification of reporter reactions specific to investigated characteristic of the disease [31]. It also facilitates the analysis of tissue‐, disease‐, or patient‐specific flux distributions, aiding in the detection of personalized metabolic responses to diseases and treatment options [40, 41]. By linking genotype to phenotype, GSMMs can identify actionable biomarkers, enabling regular screenings with less invasive methods for at‐risk groups or the general population. GSMMs have already demonstrated their utility in studying complex diseases. For example, Lewis et al. [29] classified multi‐omic data using clinical, TX, and GX inputs, along with flux distributions from multi‐omic‐constrained GSMM optimizations, to investigate radiation sensitivity in approximately 1000 samples. The study identified subgroup‐specific markers for radiation sensitivity and proposed candidate metabolite biomarkers based on flux distributions, though these remain speculative and require experimental validation.

Building on the predictive capacity and data integration potential of GSMMs, this study presents a workflow for cancer subtyping and early diagnosis, with a primary focus on lung cancer and a secondary application to PC. First, TX data are integrated into human GSMM to derive patient‐specific flux distributions (JX), capturing the dynamic metabolic states of lung cancer samples. These flux distributions, in conjunction with GX, proteomic (PX), and TX data, form the basis for robust multi‐omic classifiers. Leveraging ML techniques, random forest and ensemble voting, the classifiers identify key marker features across each omic layer, enabling precise differentiation of cancer subtypes and early‐stage disease. We prioritized certain markers using SHapley Additive exPlanations (SHAP) values borrowed from information theory. To further refine the model, the JX classifier is enhanced by integrating data from diverse platforms, including RNA sequencing and microarray datasets from tissue samples, and cell cultures, which improves the resolution and consistency of the detected metabolic markers. Finally, the enhanced multi‐omic workflow is applied to a PC dataset, which typically suffers from limited sample availability, demonstrating the broad applicability and robustness of this integrated approach. Overall, this methodology not only improves the accuracy of lung cancer subtyping and early diagnosis but also provides a systematic framework for discovering actionable biomarkers and target pathways. The integration of multiple omic layers with GSMM‐driven flux analysis represents a promising strategy for advancing personalized cancer diagnostics and treatment.

## 2 RESULTS

**FIGURE 1:**
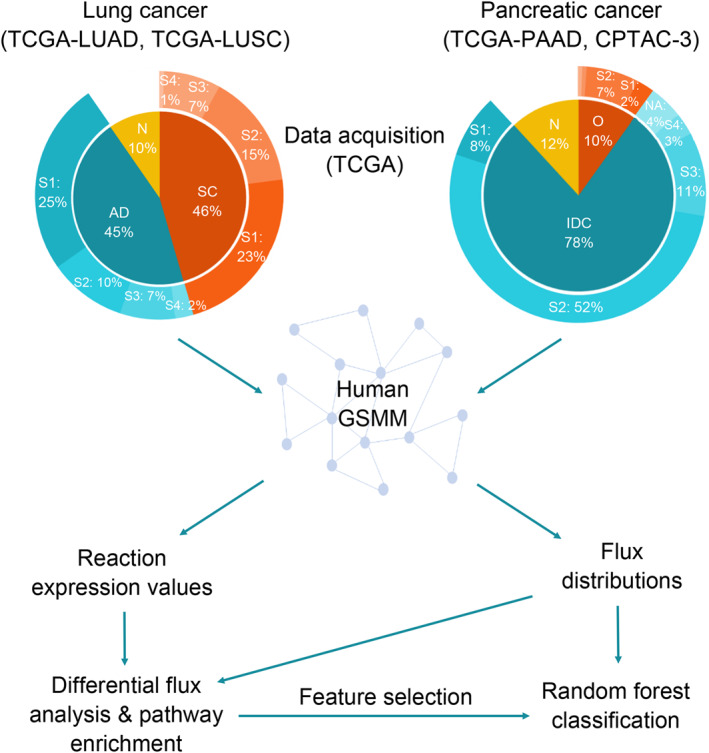
The methodology employed in this study which integrates multi‐omic data, applies metabolic modeling, and utilizes advanced statistical analysis. These approaches are combined to identify key marker pathways and features essential for the subtyping and early diagnosis of cancer.

Figure 1 outlines the methodology and details of the acquired TX data are provided in Figure S1 and Table S1 in Supporting Information S1. Initially, TX data were integrated into the human GSMM [28, 42] (8401 metabolites, 13,547 reactions, and 3268 genes) to generate sample/patient‐specific flux distributions. The performance of multi‐omic data in classifying normal and cancer samples across various subgroups and stages was evaluated. Significant features, particularly marker pathways and reactions identified through the analyses, were critically assessed to determine the added value of JX‐level data. Additionally, application of the methodology was demonstrated using the PC dataset. The findings of this study highlight the potential of this approach to pinpoint key metabolic pathways that may serve as targets for further investigation, aiding in the identification of novel, measurable biomarkers. Additionally, the methodology demonstrates its applicability to systems‐level analyses of diseases with poorly understood underlying mechanisms.

### 2.1 Generation, refinement, and pathway enrichment of fluxomic data

Sample‐specific flux distributions were generated by integrating TX data into irreversible Recon3D GSMM via the E‐flux method. Upper and lower bounds were assigned to 7280 reactions benefiting available gene–protein–reaction (GPR) rules. Several methodological refinements such as the utilization of various objective functions, data sampling methods, normalization techniques, and workflows, were implemented to maximize both classification performance and the biological relevance of the fluxome outputs. First, to minimize batch‐related variability [43], fragments per kilo base of transcript per million mapped fragments‐upper quartile (FPKM‐uq) expression data were normalized using a min–max approach anchored by the highly expressed pyruvate kinase (PKM) gene, a reference previously validated in the literature [44, 45]. This normalization approach minimized variability introduced by batch effects, improving data consistency and comparability. The biological relevance of the model outputs was confirmed by comparing the results of differential flux and gene expression analyses for each reaction with the known “hallmarks of cancer.” Additionally, at later stages, the classification performance of datasets created using various combinations of the aforementioned methods was investigated to ensure optimal results.

**FIGURE 2:**
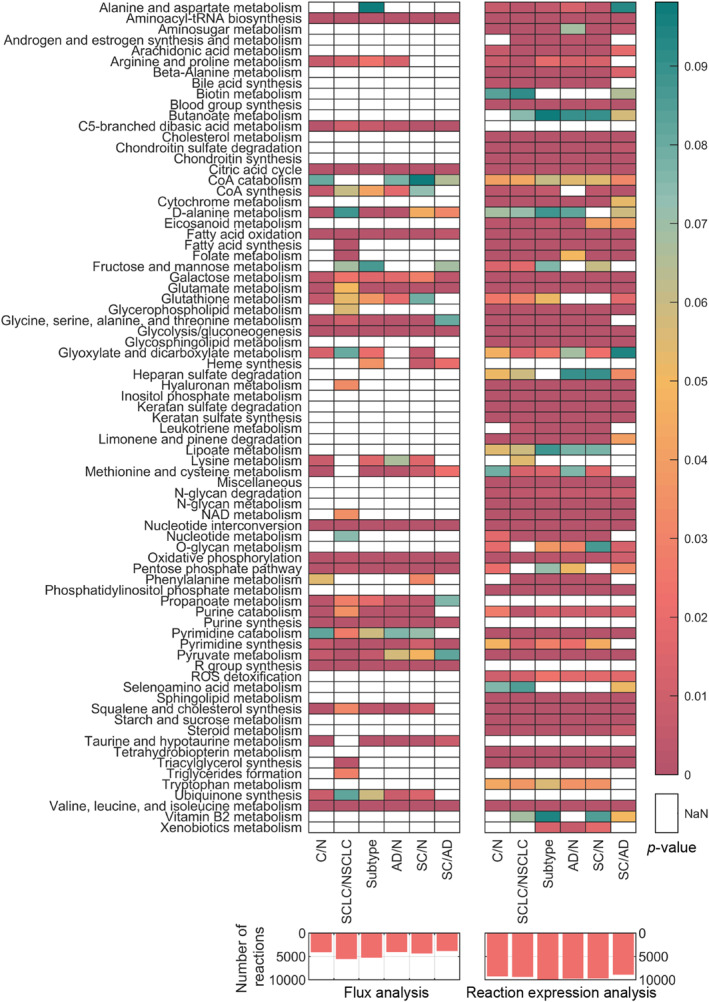
Results of the differential flux and pathway enrichment analysis of the TCGA dataset. Over‐represented subsystem terms for classification groups are displayed in heatmaps, while the number of reactions with significantly different flux and reaction expression values are illustrated in bar charts.

Following these refinements, reactions exhibiting significant differences in both flux values and their related gene expression levels across analysis groups were identified, and pathway enrichment analysis was performed. The comparative results for the cancer genome atlas (TCGA) data are presented in Figure 2, showing the number of reactions with statistically significant changes in flux values and reaction gene expression levels between conditions, as defined across six distinct statistical analyses (bar charts). Additionally, heatmaps illustrate the pathway enrichment analysis results for these reactions. As shown in Figure S2, the analysis was initially performed for all 36 comparison groups defined in the methodology. However, these early analyses did not yield notable differences, with only a small number of reactions showing significant changes between stages. As a result, the initial pathway enrichment analyses did not provide reliable results or establish clear classification patterns between stages.

Of the 117 pathways/subsystems defined in the model, 42 were found to be over‐represented in at least one group based on differential flux analysis, while 77 were over‐represented according to reaction expression analysis as shown in Figure 2. Significant changes were observed in pathways involved in glycolysis, nucleotide metabolism, amino acid metabolism, and lipid/fatty acid metabolism. These pathways are integral to cancer cell metabolism and align closely with several “hallmarks of cancer,” including sustained proliferative signaling, deregulated energy metabolism, and evasion of apoptosis [13, 46].

Additionally, triacylglycerol synthesis and fatty acid synthesis exhibited significant differences between small cell lung cancer (SCLC) and nonsmall cell lung cancer (NSCLC) subgroups, reflecting their distinct metabolic reprogramming. In comparisons between squamous cell carcinoma (SC) and adenocarcinoma (AD), only 23 pathways were over‐represented. Interestingly, pathways involved in arginine, glutathione, lysine, squalene, and ubiquinone metabolism were not significantly over‐represented in SC versus AD comparisons, suggesting subtype‐specific metabolic distinctions.

**FIGURE 3:**
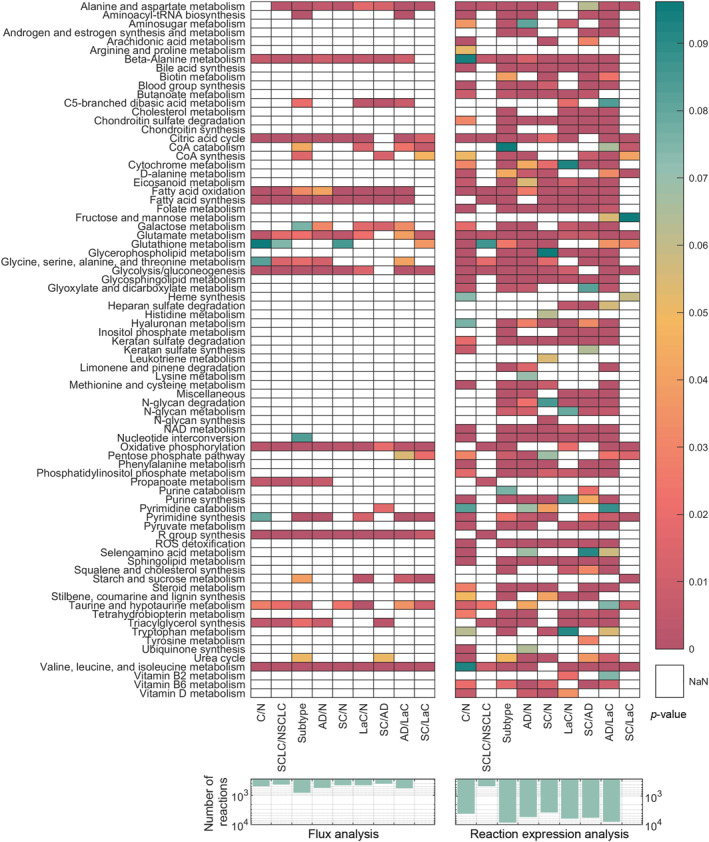
Results of the differential flux and pathway enrichment analysis of the NCBI‐GEO dataset. Over‐represented subsystem terms for classification groups are displayed in heatmaps, while the number of reactions with significantly different flux and reaction expression values are illustrated in bar charts.

Raw microarray tissue sample gene expression datasets were downloaded from the “National Center for Biotechnology Information—Gene Expression Omnibus” (NCBI‐GEO) [47] database and normalized using the robust multi‐array average method [48, 49] and min–max method using PKM gene as reference. The normalized outputs were then integrated into the model using the pipeline. Changes were identified in key metabolic processes, including central carbon and energy metabolism, amino acid and lipid metabolism, and glutathione metabolism, as illustrated in Figure 3. Of the 117 subsystems analyzed, 79 were found to be over‐represented in the reaction expression set, with significant differential expression observed between at least one pair of analysis groups. Consistent with the results of the TCGA dataset analysis, the fewest significant differences were detected in comparisons between SCLC/NSCLC and SC/LaC (large cell carcinoma). In contrast, only 24 of the 117 subsystems were significantly affected at the fluxome level, predominantly involving pathways related to central carbon and energy metabolism, fatty acid metabolism, and amino acid metabolism. Notably, a distinct pattern emerged in SC/AD comparisons, where glycolysis, taurine, pyrimidine, and glutamate metabolism pathways were not over‐represented, unlike in other group comparisons.

To evaluate the impact of differences due to sample heterogeneity, samples of different origin was integrated using the same workflow. Tissue culture samples, obtained from the “Cancer Cell Line Encyclopedia” (CCLE) [50] database, underwent the same normalization, simulation, and statistical analysis procedures as patient datasets to ensure comparability. The results of the differential flux and pathway enrichment analyses, derived from the flux distributions generated during the simulations, are presented in Figure S3. Compared with other datasets, a significantly smaller number of differentially represented pathways and reactions were identified.

### 2.2 Development of multi‐omic classifier for lung cancer

Contribution of individual omic‐layer classification models on subtyping and early diagnosis of lung cancer was evaluated by developing random forest classifiers for genomic (GX: copy number variations [CNV]), transcriptomic (TX: RNAseq), proteomic (PX: reverse‐phase protein array [RPPA]), and JX layers, allowing assessment of baseline raw data performance. We exclusively selected TCGA samples that contained comprehensive multi‐omic data to train the multi‐omic classifier. A voting classifier was then implemented, combining predictions from all omic‐layer classifications for each test sample, and performance metrics were compared across classification levels. Based on the availability of multi‐omic data, 1119 samples were used for C/N (cancer/normal) classification, 682 for AD/SC (subtype differentiation) classification, and 443 for S1/N (early‐stage) classification. GX, TX, and JX data contained approximately 20,000 features, while PX data comprised 223 features. For each dataset, subgroups were divided into 80% training and 20% test data to ensure robust model evaluation.

**FIGURE 4:**
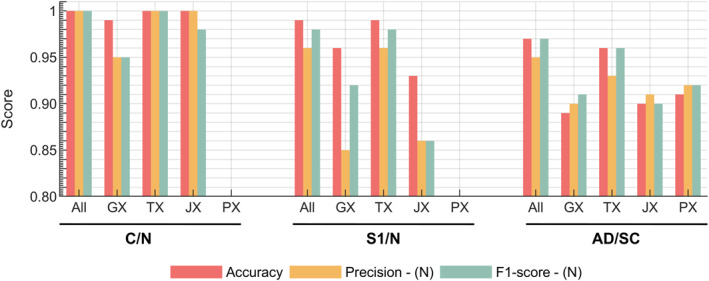
Performance of single‐omic classifiers (GX, TX, JX, PX) compared to the integrative multi‐omic classifier (“All”) across various prediction metrics, including accuracy, precision, and F1‐score for lung cancer. The analysis highlights classification scenarios for cancer versus normal (C/N), early‐stage (S1/N), and subtype differentiation (AD/SC). AD, adenocarcinoma; GX, genomic; JX, fluxomic; PX, proteomic; SC, squamous cell carcinoma; TX, transcriptomic.

Figure 4 illustrates the contributions of individual omic subgroups to prediction metrics within the multi‐omic modeling framework. It also demonstrates the performance of the multi‐omic classifier compared to single‐omic models. While the multi‐omic classifier showed comparable results to the TX classifier, it achieved slight yet notable improvements in subtyping metrics. In the C/N classification, the JX classifier performed similarly to TX, surpassing other omic layers. However, the JX models showed slightly lower performance than TX and GX for early‐stage detection (S1/N classification). The multi‐omic classifier reached perfect accuracy for in C/N classification, maintaining high accuracy despite minor reductions in precision and F1‐score for TX. Additionally, while JX outperformed GX in C/N classification, a slight dip in F1‐score indicated fewer true positive predictions.

For S1/N classification, which evaluates early‐stage detection, multi‐omic classifiers achieved metrics exceeding 0.95. GX and JX classifiers showed notable drops in performance, particularly with false positive predictions, with F1‐scores demonstrating the highest variability. TX models consistently displayed significantly higher precision across classifications. Subtype classifiers (SC/AD) reached accuracy levels above 0.9 but were lower than other classifications, with TX slightly outperforming other omic layers.

**FIGURE 5:**
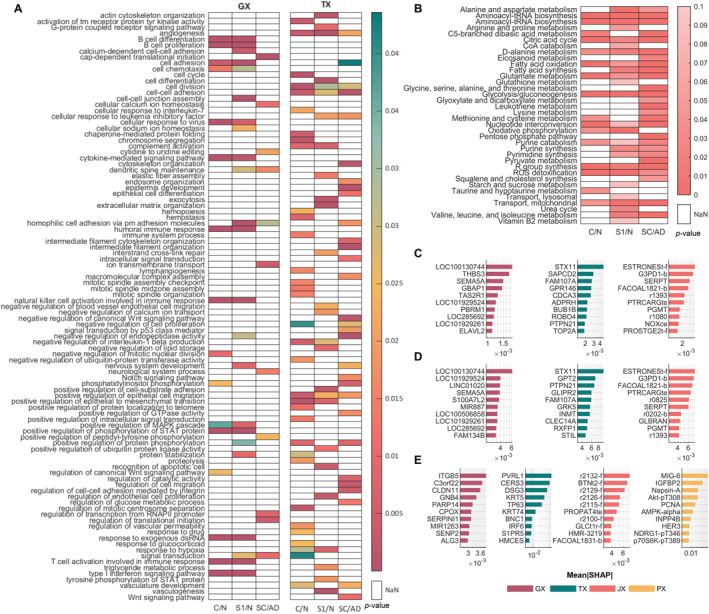
Functional analyses of features with high importance (mean|SHAP| > 1e‐4) across all individual omic layers. (A) GO analysis results for GX‐ and TX‐level features, presented as heatmaps showing *p*‐values for the representation of GO bioprocess terms across three classification groups: C/N, S1/N, and AD/SC. (B) Pathway enrichment analysis results for JX‐level features, highlighting the metabolic pathways enriched in each classification group. (C–E) Names and mean|SHAP| values of the top 10 reactions for C/N (C), S1/N (D), and AD/SC (E) classifications, respectively, showcasing the reactions with the highest importance in distinguishing these groups. AD, adenocarcinoma; GO, Gene Ontology; GX, genomic; JX, fluxomic; SC, squamous cell carcinoma; TX, transcriptomic.

Key marker processes and features for the classification groups are shown in Figure 5. Similar pathways identified in earlier analyses are highlighted, with detailed features for classification provided in Table S2.

The integrative analysis yielded distinct metabolic flux signatures across cancer types. Features with the highest SHAP values were identified across multiple omic layers. For instance, in the GX layer, markers such as THBS3 (Thrombospondin 3) and SEMA5A (Semaphorin 5A) were prominent; these molecules are known to mediate cell–matrix adhesion and angiogenesis, processes that are critical in NSCLC. In addition, GBAP1, involved in sphingolipid metabolism and autophagy, and PBRM1, a regulator of the cell cycle and chromatin remodeling, were detected, supporting the established literature linking these pathways to lung tumor progression. Complementary to these, TX markers including STX11, SAPCD2, FAM107A, GPR146, and CDCA3 arose, underscoring roles in protein transport, cell division, growth, and ubiquitination. Moreover, JX‐level data contributed metabolic markers such as G3PD1 (glycerol‐3‐phosphate dehydrogenase) and SERPT (serine C‐palmitoyltransferase), which reflect reprogramming of lipid and carbohydrate metabolism. A set of noncoding RNAs (including LOC100130744, LINC01020, S100A7L2, and MIR887) further extended the candidate pool, providing prospects for novel diagnostic biomarkers.

Aligning with the canonical Warburg effect and the anabolic demands of cancer cells, altered expression levels of metabolic enzymes such as G3PD1 and regulators such as THBS3 and SEMA5A indicate a shift toward enhanced glycolysis and modified extracellular matrix interactions in the C/N comparison [51]. Moreover, among lung cancer subtypes, the differential expression of markers such as ITGB5, CLDN11, and KRT5 between AD and SC underscores subtype‐specific metabolic adaptations that affect cell adhesion and signaling pathways. Complementing these findings, the S1/N analysis, capturing non‐coding RNAs and post‐transcriptional modulators (e.g., LOC100130744, LINC01020, S100A7L2, MIR887), suggests that fine‐tuning of metabolic pathways by RNA‐mediated regulation plays an additional role in driving the altered phenotypes observed in lung tumors.

### 2.3 Improving JX classifier performance for lung cancer

To further enhance the efficacy of JX data from different platforms in distinguishing subtypes and stages individually, various ML models were developed using combinations of sampling techniques, data types, classification algorithms, and normalization/standardization methods. Classification models were applied separately to JX data obtained from patient samples (via RNA‐seq and microarray) and tissue culture samples (from CCLE) to assess their individual performance without a priori feature selection. Among the tested models, random forest classification was identified as the most efficient, achieving superior metrics when applied to fluxome data [52, 53]. For instance, Figure S4 illustrates that the support vector machines classifiers (SVM) underperformed relative to the random forest models, with performance metrics dropping by 10%–20% in several instances. For each dataset, subgroups were divided into 80% training and 20% test and classifier parameters were optimized by GridSearchCV [54] function searching exhaustively over a specified parameter grid.

The sensitivity and accuracy metrics for the models are presented in Table 1, demonstrating their ability to correctly identify true positive cases and classify samples with high accuracy. The receiver operating characteristic (ROC) curves, displayed in Figure S5, provide a graphical representation of the true positive rate versus the false positive rate for each model, offering insights into their diagnostic performance. Additionally, Figure S6 presents the ROC curves for the classification of AD and SC stages, highlighting the trade‐off between sensitivity and specificity for each model. Despite efforts to improve performance metrics, the classification models struggled to achieve reliable accuracy across staging categories. The area under the curve (AUC) values remained modest, with limited improvements observed even after optimization, indicating challenges in distinguishing between stages within the same subtype. This performance disparity underscores the challenge of accurately predicting cancer stage transitions.

**TABLE 1:** Performance metrics of random forest classification of the fluxomic data.

Classification group	Class label	Precision			Recall			F1‐score		
		CCLE	TCGA	NCBI	CCLE	TCGA	NCBI	CCLE	TCGA	NCBI
C/N	N	1.00	1.00	0.94	0.33	0.95	0.77	0.50	0.98	0.85
	C	0.88	0.99	0.98	1.00	1.00	1.00	0.94	0.99	0.99
NS	SCLC	1.00	1.00	1.00	1.00	1.00	0.14	1.00	1.00	0.25
	NSCLC	1.00	1.00	0.97	1.00	1.00	1.00	1.00	1.00	0.99
Subtype	AD	0.86	0.97	0.89	1.00	0.98	0.97	0.92	0.98	0.93
	SC	0.00	—	1.00	0.00	—	0.12	0.00	—	0.22
	LaC	0.00	0.98	0.85	0.00	0.98	0.68	0.00	0.98	0.76

Abbreviations: AD, adenocarcinoma; C/N, cancer vs normal; LaC, large cell carcinoma; NS: small cell lung cancer vs nonsmall cell lung cancer; NSCLC, nonsmall cell lung cancer; SC, squamous cell carcinoma; SCLC, small cell lung cancer.

The results indicate that the CCLE data, as highlighted in the statistical analysis section, did not exhibit significant separation between conditions. This lack of separation is reflected in the performance metrics of the classifiers. Specifically, for the C/N classification, the CCLE data achieved a recall of 0.33 and an F1‐score of 0.5. These metrics indicate poor performance in correctly identifying true positive cases (recall) and the balance between precision and recall (F1‐score). In contrast, other datasets demonstrated higher accuracy, with success rates of 85%–100% in distinguishing normal and cancer samples, highlighting the robustness of their classifications.

The TCGA dataset exhibits the highest performance metrics among all the datasets evaluated. Notably, it achieves scores higher than 95% across all metrics. This highlights the reliability of the TCGA dataset in accurately identifying samples with minimal error.

Conversely, the CCLE and NCBI‐GEO datasets displayed greater variability in their metrics. For instance, while CCLE achieves perfect scores in distinguishing SCLC and NSCLC categories, it shows significant decreases in recall and F1‐score when identifying N (recall: 0.33, F1: 0.50). This suggests that the CCLE dataset may encounter challenges in reliably identifying negative samples. Similarly, although the NCBI‐GEO dataset generally maintains high precision and recall across most cases, there are notable outliers. For example, in the SC category, a low recall value (0.14) results in a significantly reduced F1‐score (0.25). This indicates that while NCBI‐GEO excels in identifying true positives, it exhibits lower recall of all positive instances, thus affecting the overall balance represented by the F1‐score.

When focusing on the subtype group, AD has high and consistent performance metrics across all datasets, indicating strong reliability in identifying AD samples. However, SC and LaC subtypes reveal discrepancies. Notably, CCLE shows zero performance in both SC and LaC, indicating a complete lack of identification capability in these subtypes. NCBI‐GEO, while having perfect precision in SC, exhibits much lower recall and F1‐scores, pointing to potential issues in sample detection reliability. These findings underscore the superior consistency and reliability of the TCGA dataset compared to CCLE and NCBI‐GEO.

**FIGURE 6:**
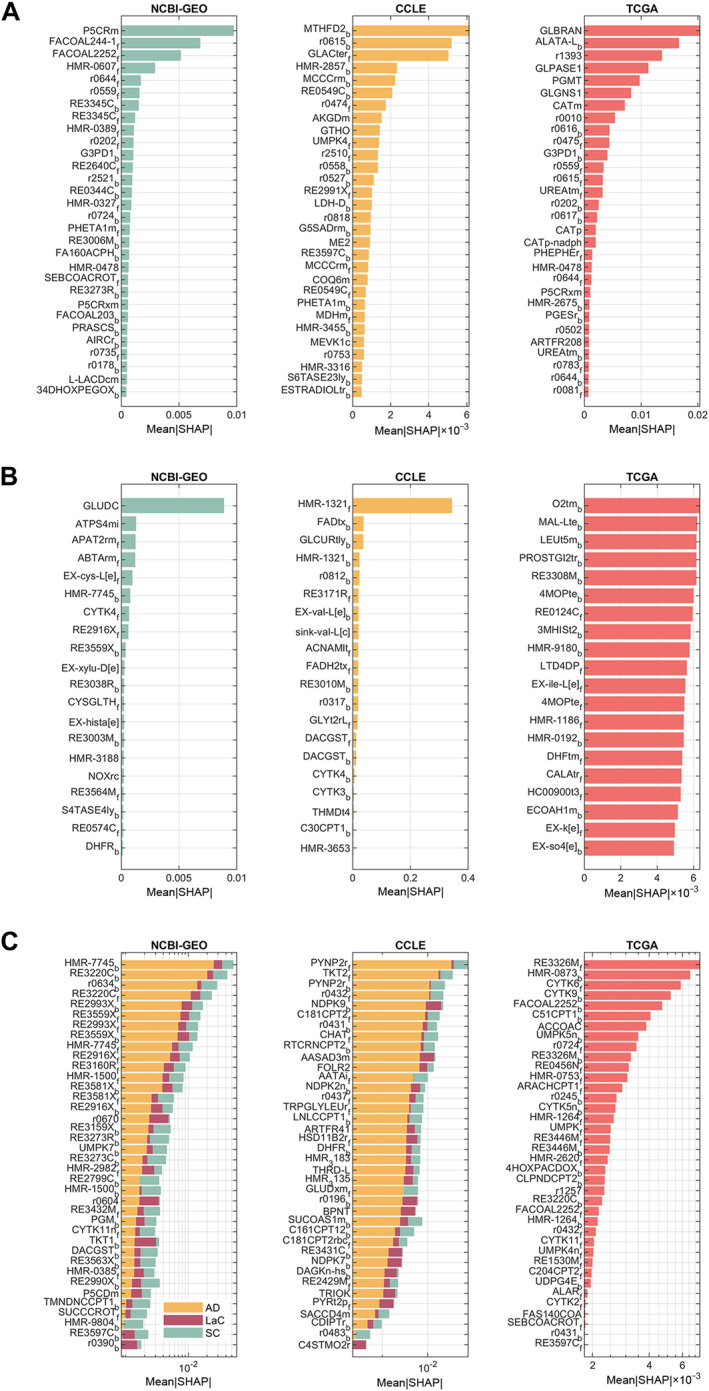
Reactions with the highest mean|SHAP| values for each dataset. Parts (A–C) display the reactions with the greatest importance in the classification of C/N, small cell lung cancer/nonsmall cell lung cancer, and subtypes, respectively.

Reactions that play a significant role in distinguishing each group included in the classification have been identified through SHAP value analysis of resulting classifiers. The results of the analysis are presented in Figure 6. Since more than two subtype data were present in NCBI‐GEO and CCLE datasets, SHAP values for features were shown for each of the different subtypes. The top reactions with the highest importance and the pathways to which they belong are shown in Table 2. The table highlights key metabolic enzymes (excluding transport reactions) grouped by datasets (GEO, TCGA, CCLE) and lung cancer classifications (C/N, SCLC/NSCLC, molecular subtypes). These enzymes play critical roles in tumor metabolism, with implications for diagnosis, subtyping, and therapy. Together, these findings weave a coherent story that highlights both known and emerging biomarkers capable of further refining our understanding and treatment of lung cancer subtypes.

**TABLE 2:** Key reactions with highest SHAP values for subgroup classification using fluxome data across different platforms, excluding transport and exchange reactions.

Group	Data	Abbreviation	Description	Subsystem
C/N	GEO	P5CRm	Pyrroline‐5‐carboxylate reductase, mitochondria	Arginine and proline metabolism
		FACOAL244_1	Fatty‐acid—CoA ligase	Fatty acid oxidation
		FACOAL2252	Fatty‐acid—CoA ligase	Fatty acid oxidation
		HMR_0607	Phosphatidate cytidylyltransferase	Glycerophospholipid metabolism
		r0644	(S)‐Methylmalonate semialdehyde:oxygen oxidoreductase	Valine, leucine and isoleucine degradation
	TCGA	GLBRAN	1, 4‐Alpha‐glucan branching enzyme (Glygn1 → Glygn2)	Starch and sucrose metabolism
		ALATA_L	L‐alanine transaminase	Glutamate metabolism
		r1393	Glycogen phosphorylase	Starch and sucrose metabolism
		GLPASE1	Glycogen phosphorylase (Glygn2 → Dxtrn)	Starch and sucrose metabolism
		PGMT	Phosphoglucomutase	Glycolysis/gluconeogenesis
SCLC/NSCLC	GEO	GLUDC	Glutamate decarboxylase	Glutamate metabolism
		ATPS4mi	ATP synthase (four protons for one ATP)	Oxidative phosphorylation
		APAT2rm	3‐Aminopropanoate:2‐oxoglutarate aminotransferase, mitochondrial	Beta‐alanine metabolism
		ABTArm	4‐Aminobutyrate transaminase, reversible, mitochondrial	Glutamate metabolism
		HMR_7745	D‐glucose 1‐epimerase	Glycolysis/gluconeogenesis
Subtypes	GEO	HMR_7745	D‐glucose 1‐epimerase	Glycolysis/gluconeogenesis
		RE3220C	Steryl‐sulfatase	Androgen and estrogen synthesis and metabolism
		r0634	Octanoyl coenzyme A:acetyl coenzyme A C‐acyltransferase	Fatty acid oxidation
		RE2993X	2, 4‐Dienoyl coenzyme A reductase (NADPH)	Fatty acid oxidation
		RE3559X	Acetyl coenzyme A C‐acyltransferase	Eicosanoid metabolism
	CCLE	PYNP2r	Pyrimidine‐nucleoside phosphorylase (Uracil)	Pyrimidine catabolism
		TKT2	Transketolase	Pentose phosphate pathway
		r0432	Palmitoyl coenzyme A:L‐carnitine O‐palmitoyltransferase	Fatty acid oxidation
		NDPK9	Nucleoside‐diphosphate kinase (ATP:IDP)	Nucleotide interconversion
		C181CPT2	Transport of octadecenoyl coenzyme A into mitochondrial matrix	Fatty acid oxidation
	TCGA	RE3326M	RE3326M	Valine, leucine, and isoleucine metabolism
		HMR_0873	3‐Galactosyl‐N‐acetylglucosaminide 4‐alpha‐L‐fucosyltransferase	Glycosphingolipid metabolism
		CYTK6	Cytidylate kinase (CMP, CTP)	Nucleotide interconversion
		CYTK9	Cytidylate kinase (CMP, dCTP)	Nucleotide interconversion
		FACOAL2252	Fatty‐acid—coenzyme A ligase	Fatty acid oxidation

Abbreviations: CCLE, Cancer Cell Line Encyclopedia; GEO, Gene Expression Omnibus; NSCLC, nonsmall cell lung cancer; SCLC, small cell lung cancer.

#### 2.3.1 C/N classification

Metabolic shifts between tumor and nontumor tissues in the C/N group reveal significant metabolic rewiring in tumor tissues. For instance, pyrroline‐5‐carboxylate reductase (PYCR1), essential for proline synthesis, emerged as a pivotal enzyme promoting tumor growth, collagen remodeling, and redox balance, factors linked to poor prognosis [55]. In lung cancer, PYCR1 activity precedes lipid oxidation via fatty‐acid‐CoA ligases (FACOAL244_1 and FACOAL2252), essential for energy production and overexpressed in KRAS‐mutant NSCLC, which relies on fatty acid oxidation (FAO) for survival. This sequential activation not only validates the classifier’s ability to capture known metabolic shifts but also opens avenues for subtype stratification and therapeutic intervention using FAO inhibitors, such as etomoxir [56, 57]. Complementing these findings, phosphatidate cytidylyltransferase (HMR_0607) was found to be upregulated in EGFR‐mutant lung adenocarcinoma (LUAD) and is linked to resistance against EGFR‐targeted therapies, thus positioning it as a promising companion diagnostic marker [58, 59].

Moreover, the enzyme (S)‐methylmalonate semialdehyde: oxygen oxidoreductase (r0644), a key player in branched‐chain amino acid (BCAA) degradation, supports energy production and biosynthesis. Dysregulated BCAA metabolism correlates with poor prognosis in NSCLC, making r0644 activity a potential biomarker for tumors with metabolic plasticity under stress [60, 61]. As another amino acid metabolism related reaction, L‐alanine transaminase (ALATA_L) was shown to facilitate the alanine‐pyruvate interconversion, thereby maintaining redox balance and fueling biosynthetic demands while contributing to immune evasion. This is further supported by the predictive utility of the aspartate aminotransferase/alanine aminotransferase (AST/ALT) ratio in NSCLC survival outcomes [62].

Finally, TCGA‐trained classifiers also underscored the significance of glycogen metabolism. Key regulators, including 1,4‐alpha‐glucan branching enzyme (GLBRAN), glycogen phosphorylase (GLPASE1), and phosphoglucomutase, were identified as modulators of glycogen storage and utilization, underpinning glycogen‐dependent survival mechanisms in LUAD. Their involvement in chemoresistance and glycolysis‐independent strategies not only reinforces their potential as biomarkers but also as therapeutic targets when combined with conventional treatments [63].

#### 2.3.2 SCLC/NSCLC classification

The SCLC/NSCLC group emphasizes reactions participating in glutamate metabolism, oxidative phosphorylation, and beta‐alanine metabolism, pointing to their roles in neurotransmitter synthesis and energy production. For example, glutamate decarboxylase, an enzyme critical to GABAergic signaling, was reported to be upregulated in SCLC. This pathway fosters tumor growth and immune evasion, demonstrating its potential to distinguish neuroendocrine SCLC from epithelial NSCLC [64]. Similarly, ATP synthase (ATPS4mi), a key component of oxidative phosphorylation, is known to show elevated expression in chemotherapy‐resistant SCLC, suggesting its inhibition as a strategy to overcome resistance [65]. Additionally, 3‐aminopropanoate:2‐oxoglutarate aminotransferase (APAT2rm) is linked to β‐alanine metabolism and the tricarboxylic acid (TCA) cycle, serving as a crucial metabolic intersection. Although studies on its role in lung cancer are limited, its activity aligns with findings in broader cancer contexts, such as regulating osmotic balance, vitamin B5 biosynthesis, and redox balance. The mitochondrial enzyme 4‐aminobutyrate transaminase (ABTArm), which regulates the gamma‐aminobutyrate (GABA) shunt, is associated with immune‐cold microenvironments in SCLC, further contributing to immune evasive mechanisms [66]. Lastly, D‐glucose 1‐epimerase (HMR_7745), a glycolytic enzyme, supports alterations in glucose metabolism, with its role in lung cancer metabolism requiring further exploration.

#### 2.3.3 AD/SC classification

In AD/SC analysis, markers identified across multiple datasets reveal distinct metabolic rewiring between subtypes. Alterations across key pathways such as FAO, glycolysis/gluconeogenesis, nucleotide interconversion, and pyrimidine catabolism were observed. For example, in glucose metabolism, D‐glucose 1‐epimerase (HMR_7745) was highlighted again. Complementarily, transketolase in the pentose phosphate pathway provides nucleotide biosynthesis precursors and maintains redox balance; its overexpression in aggressive AD was reported to be correlated with poor prognosis [67]. In the context of lipid metabolism, another notable feature, palmitoyl coenzyme A:L‐carnitine O‐palmitoyltransferase (r0432), facilitates mitochondrial FAO, a process critical to energy production in metabolically plastic tumor types such as SC. Transport reaction of octadecenoyl coenzyme A into the mitochondrial matrix (C181CPT2) highlights the role of mitochondrial transport systems in facilitating fatty acid metabolism, a hallmark of certain lung cancer subtypes. In addition, enzymes such as octanoyl coenzyme A:acetyl coenzyme A C‐acyltransferase (r0634) [68] and 2,4‐dienoyl coenzyme A reductase (RE2993X) play integral roles in FAO, which is frequently utilized by aggressive SC tumors. Furthermore, acetyl coenzyme A C‐acyltransferase (RE3559X) plays a pivotal role in eicosanoid metabolism and lipid biosynthesis, processes often dysregulated in cancer. Steryl‐sulfatase (RE3220C), involved in androgen and estrogen metabolism, offers another layer of complexity as hormonal influences may modulate its activity in certain lung cancer contexts [69]. For pyrimidine metabolism, pyrimidine‐nucleoside phosphorylase (PYNP2r) participates in nucleotide salvage and DNA repair, pathways critical for the survival of rapidly proliferating tumor cells. Nucleoside‐diphosphate kinase, a key enzyme in nucleotide interconversion, supports nucleotide biosynthesis and signaling processes essential for tumor growth and metastasis, with potential implications for both AD and SC. These features have scarce information about how they differentiate between different subtypes, highlighting their potential as biomarkers for distinguishing between subtypes.

### 2.4 Application in pancreatic cancer dataset

PC data were utilized to evaluate the performance of the multi‐omic classification and pathway enrichment methodology on smaller sample sizes. Specifically, 161 samples from TCGA‐PAAD/CPTAC3 datasets were included for the C/N classification, while 138 samples were used for the S1/N classification. All omic data exhibited similar feature distributions to lung cancer omic data, ensuring consistency in the analytical framework.

**FIGURE 7:**
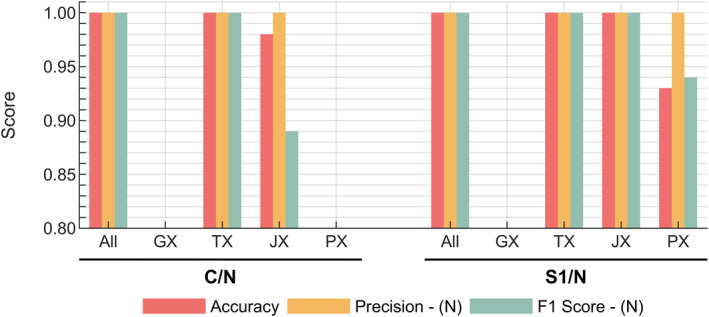
Performance of single‐omic classifiers (transcriptomic, fluxomic, proteomic) compared to the integrative multi‐omic classifier (“All”) across various prediction metrics, including accuracy, precision, and F1‐score for pancreatic cancer. The analysis highlights classification scenarios for cancer versus normal (C/N) and early‐stage differentiation.

Figure 7 illustrates the contributions of individual omic subgroups to prediction metrics within the multi‐omic modeling framework for PC data. The analysis reveals that both TX and JX classifiers demonstrated comparable performance, with JX showing slightly lower true positive classification rates compared to TX. In the S1/N classification, multi‐omic, TX, and JX models achieved perfect scores, while PX exhibited a decline in true positive rate. Despite some variability across classifiers in specific metrics, all models maintained an accuracy of at least 85% in distinguishing between relevant groups. Overall, the multi‐omic classification methodology proved effective for PC.

**FIGURE 8:**
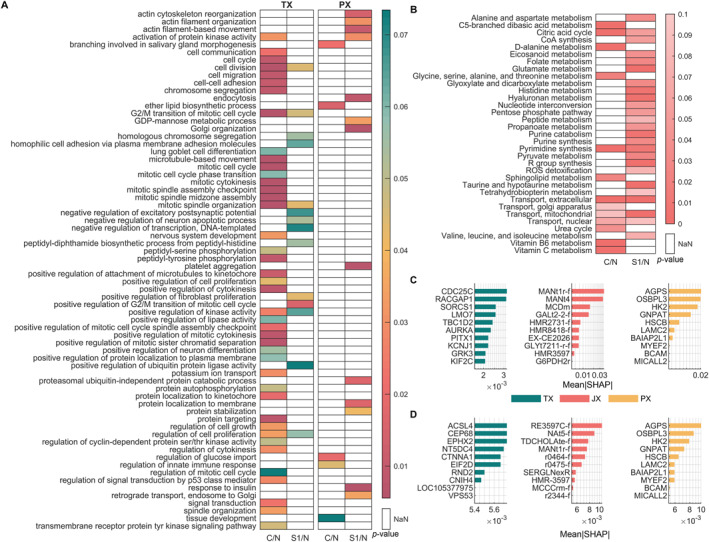
Functional analyses of features with high importance (mean|SHAP| > 1e‐4) across all individual omic layers for pancreatic cancer. (A) Gene Ontology analysis results for transcriptomic‐ and proteomic‐level features, presented as heatmaps showing *p*‐values for the representation of GO bioprocess terms across three classification groups: C/N and S1/N. (B) Pathway enrichment analysis results for fluxomic‐level features, highlighting the metabolic pathways enriched in each classification group. (C and D) Names and mean|SHAP| values of the top 10 reactions for C/N (C) and S1/N (D) classifications, respectively, showcasing the reactions with the highest importance in distinguishing these groups.

Following the detection of important features of classifications, functional enrichment analyses were performed for each omic level as explained before for lung cancer. Results of the functional analyses for each omic‐layer and features of high importance were shown in Figure 8. The details of the detected features were explained in Table S3. Together, these diverse features across omic layers reflect both the established metabolic alterations in PC and novel insights that could extend current biomarker panels.

Functional enrichment analysis of multi‐omic data in PC has revealed a spectrum of biomarkers, both established and novel, that underscore critical oncogenic processes. In C/N classifications, transcript‐level regulators such as CDC25C and RACGAP1 emerge as pivotal: CDC25C activates the cyclin B1/CDK1 complex to drive the G2/M transition and was shown to be overexpressed in PC, correlating with tumor proliferation and poor prognosis [70], while RACGAP1—by modulating cytokinesis and oncogenic signaling—has prognostic implications linked to immune evasion and therapy resistance [71, 72]. Novel transcript candidates, including SORCS1, LMO7, and TBC1D2, further enrich this narrative. SORCS1, involved in vesicle‐mediated transport, may influence metabolic regulation [73]; LMO7, a LIM domain protein, drives tumor progression and metastasis through effects on cell‐cycle arrest and apoptosis [74, 75]; and TBC1D2, a Rab7 GTPase regulator, modulates endocytic trafficking, thereby contributing to PC aggressiveness [76].

In the joint JX and PX layers, metabolic enzymes such as HK2 (hexokinase 2), AGPS (alkylglycerone phosphate synthase), OSBPL3 (oxysterol binding protein‐like 3), and GNPAT (glyceronephosphate O‐acyltransferase) underscore the metabolic rewiring characteristic of PC. HK2, a key glycolytic enzyme, is overexpressed in PC and correlates with poor prognosis, emphasizing its role in energy production and tumor growth [77]. AGPS and OSBPL3 are involved in lipid metabolism, with OSBPL3 showing prognostic significance in PC [78], while GNPAT supports lipid biosynthesis and cellular homeostasis. New insights include MCDm, which underpins FAO; MANt4 [79], facilitating D‐mannose transport and potentially modulating glycolysis; and G6PDH2r, a central player in the pentose phosphate pathway driving redox balance and anabolic growth [80, 81]. These metabolic enzymes are pivotal in supporting PC’s hallmark rewired energy production. Additionally, features such as BAIAP2L1, MICALL2, and BCAM emphasize the importance of cytoskeletal dynamics, cell adhesion, and angiogenesis; MICALL2, for instance, maintains epithelial integrity yet can drive metastasis when dysregulated [82, 83], while BCAM [84] and LAMC2 [85] are integral to tumor invasion and extracellular matrix interactions. Although HSCB and MYEF2 are less characterized, their roles in mitochondrial integrity and transcriptional regulation, respectively. Given their roles in mitochondrial integrity and transcriptional regulation respectively, they are good candidates for further exploration.

For early diagnosis of PC, analyses revealed a network of markers spanning several key metabolic pathways. ACSL4, a pivotal enzyme in long‐chain fatty acid metabolism, has been linked to ferroptosis—a form of regulated cell death driven by lipid peroxidation. Its dysregulation in PC not only supports tumor growth but also underlies resistance to oxidative stress, making it a promising candidate for early diagnosis and therapeutic intervention [86]. In parallel, CEP68 plays an essential role in centrosome cohesion and cell cycle regulation, with its disruption contributing to GX instability and aggressive tumor phenotypes [87]. EPHX2, on the other hand, regulates lipid metabolism by promoting fatty acid degradation, thereby inhibiting cancer progression and underscoring its potential as a biomarker in a cancer marked by extensive lipid rewiring [88].

Subsequently, CTNNA1, encoding α‐catenin, is integral to cell adhesion and the E‐cadherin‐catenin complex. Dysregulation of CTNNA1 disrupts adherent junctions, facilitating tumor invasion and metastasis. Its role in maintaining epithelial integrity underscores its potential as a diagnostic marker for PC [89]. Carbonyl reductase modulates inflammatory responses via eicosanoid metabolism, where elevated eicosanoid production is tied to PC progression [90]. Additionally, 4‐aminobutyraldehyde:NAD^+^ oxidoreductase contributes to redox balance and energy production through its role in glutamate metabolism. Enzymes involved in nucleotide and amino acid metabolism also emerge as key players; 2‐deoxyadenosine 5‐diphosphate:oxidized‐thioredoxin 2‐oxidoreductase is crucial for DNA synthesis and repair, while the L‐serine/glutamine reversible antiport highlights the dependency of cancer cells on glutamine for biosynthesis and energy [91]. Finally, methylcrotonoyl coenzyme A carboxylase, involved in BCAA metabolism, reflects the metabolic shifts characteristic of aggressive tumors. Together, these markers not only delineate the metabolic landscape of PC but also offer promising targets for early diagnosis and intervention.

In conclusion, the constructed pipeline effectively integrates multi‐omic data to achieve high accuracy levels across various cancer classifications. By leveraging omic‐layer classifiers and baseline models, the study highlights key biological pathways and features that contribute to classification precision, particularly in early‐stage detection of both lung and PCs.

## 3 DISCUSSION

In this study, we explored the subtyping and early diagnosis of lung and PCs, two diseases notoriously difficult to detect at early stages, by integrating published multi‐omic data with sample‐specific flux profiles derived through TX‐constrained GSMM simulations. This methodology translates TX‐level data into functional metabolic alterations, helping to uncover key pathways and reactions that distinguish cancer subtypes and disease stages. Moreover, the approach holds promise for less characterized diseases, as building disease‐specific GSMMs and integrating diverse omic layers can reveal unique molecular signatures that inform ML classifiers for early disease‐ or subtype‐specific predictions.

The results shown in Figures 2 and 3, demonstrate that the proposed methodology successfully reflects cancer metabolism. Within the framework of the “hallmarks of cancer,” literature indicates changes in energy metabolism, glutaminolysis, lipid metabolism, amino acid metabolism, and mitochondrial metabolism [92]. Tumor cells, driven by rapid growth, turn to alternative energy sources such as glutamine, serine, glycine, and various carbohydrate sources [13]. Furthermore, the cellular energetic machinery shifts toward aerobic glycolysis and increased respiration to obtain the necessary energy for growth. To support these increased tendencies for growth and survival, the production of signaling precursors and cellular structure metabolites within lipid metabolism becomes crucial [46].

Nevertheless, obtaining flux distributions has two main limitations. One group of limitations stems from the nature of the samples/data, while the other arises from the process of reflecting these differences between samples in simulation through flux analysis methods. First, intrinsic sample heterogeneity and clinical and lifestyle differences (dietary habits, presence or absence of comorbidities, smoking status, etc.) create variability in TX profiles that may confound disease‐specific alterations. We attempted to mitigate this by emphasizing robust pathway‐level analysis (comparison with the “hallmarks of cancer”) and using FPKM‐uq data with min–max normalization, guided by stable reference genes like PKM, to counter batch effects [45]. A systematic evaluation of objective functions, normalization techniques ensured the selection of datasets that achieved optimal classification performance while maintaining biological relevance. Further improvements in flux distributions can be performed by identifying objective functions specific to cancer type, treatment type, or according to the feature to be investigated [28]. Second, flux analysis is complicated by nonunique solutions and branching differences. Although model reduction using pruning methods (GIM3E) [41] and Euclidean norm minimization via the E‐flux approach [93] were employed, simulation times were considerable (8–10 h per sample) and improvements in classification performance were marginal when 45 randomly selected sample TX data from different subtypes were analyzed (data not shown). Consequently, efforts to achieve unique solutions were halted. The relevance of the flux distributions was assessed using manual reduction of futile cycles and literature‐supported analysis of active and inactive reactions and marker pathways. Although this methodology effectively captured metabolic alterations, the direct correlation between TX data and enzyme activity, remain oversimplified, but it is still valid. In this study, transcriptome data was used as it was the most widely available across groups. If proteome data had been equally accessible, it could have been utilized as well. This assumption can be further addressed by the use of protein‐constrained or kinetic models supported by dedicated ^13^C metabolic flux analysis [94] of marker pathways in the presence of related data [28].

Despite the refinements, certain flux changes appeared to result from mathematical artifacts or individual sample variability, limiting pathway enrichment analyses for staging classifications as shown in Figures S2 and S6 in Supporting Information S1. The staging analysis revealed that differences between cancer and normal samples, as well as between subtypes, were much more pronounced than those between stages. The limited number of significantly altered reactions between stages and potential mathematical artifacts (e.g., futile cycles, transport reaction variances) suggest that staging classification requires further refinement. Although stage differences were captured with 60%–70% accuracy when classification was performed, attempts at staging were not continued. This was also evident in applications conducted using CCLE tissue culture data (Figure S3) possibly due to difficulties in capturing the differences when comparing highly homogenous CCLE tissue culture data, which performed less robustly, instead of comparing heterogeneous patient samples.

ML–aided multi‐omics integration has emerged as a valuable tool in cancer research [23, 29, 95, 96]. By combining GX, TX, and PX data, our approach exposes intricate interactions and biomarkers that single‐omics strategies might miss [96]. Additionally, GSMMs enable detailed analyses of how alterations at these omic levels translate into cellular metabolism, providing valuable insights into how molecular changes generate precursors for essential cellular processes [28, 97, 98]. As illustrated in Figures 5 and 8, most significantly altered features at GX and TX level were associated with cellular structure formation, cell cycle regulation, or signaling pathways—hallmarks of cancer [92, 99]. Yet, the heterogeneity of tumor specimens often obscures cancer‐specific changes; hence, our focus on flux‐level analysis enables us to detect metabolic markers with more direct clinical relevance. It was demonstrated that a random forest classifier can effectively subtype and detect early‐stage cancer. Among several models tested, random forest was selected for its superior performance, displaying high precision for cancer/normal (C/N) classification and robust consistency across omic layers (Figure S4) [52, 53]. The methodology’s success in achieving high classification performance for both cancer types, highlights its robustness. Perfect classification scores were observed when all omic layers were included (Figures 4 and 7), emphasizing the benefit of a comprehensive multi‐omic approach. The individual performances of the omic‐layer models showed that the fluxome and transcriptome data provided similar results in the C/N classification, whereas the early diagnosis (S1/N) of cancer types benefited more from TX and GX models. This suggests that certain omic layers may be more informative for specific classification tasks, and integrating them maximizes predictive accuracy. For lung cancer, integrating TX and JX, with additional GX and PX data, allowed for a detailed analysis. While omitting PX data in C/N and S1/N classifications slightly reduced precision and F1‐scores, the overall performance remained high. This demonstrates the method’s effectiveness in handling large datasets and underscores the need for comprehensive data integration. For PC, the inclusion of multi‐omic data samples showed high performance similar to lung cancer with TX and JX. This indicates that even with smaller sample sizes, the method can provide reliable results. The high precision and robust overall performance of the multi‐omic voting classifier emphasize that integration of diverse data types can mitigate the limitations inherent to individual omic analyses, despite slight reductions in F1‐scores and recall, particularly in early‐stage detection, indicate that further refinements are necessary. These performance variabilities, likely due to class imbalance and inherent dataset differences, calls for future work that includes improved model tuning and data augmentation.

This integrated multi‐omics approach underscores the metabolic complexity and heterogeneity of lung cancer by both validating established biomarkers and uncovering novel candidates for further functional and clinical exploration (Table S2, Figure 5). C/N analyses confirm that hallmark metabolic reprogramming, such as enhanced glycolysis, increased lipid biosynthesis, and a general boost in biosynthetic capacity, is critical for tumor proliferation under nutrient‐limited conditions. Subtype comparisons (AD/SC) reveal that genetic and histopathological differences drive distinct metabolic adaptations, including changes in cell adhesion and integrin‐mediated signaling, which correlate with differences in clinical behavior. Significantly, the integration of early‐stage (S1/N) layers emphasizes the roles of noncoding RNAs and post‐transcriptional regulators as key modulators of metabolic pathways, offering nuanced insights into enzyme activity and flux distributions that bulk TX analyses often miss.

Moreover, individual classifiers demonstrate that while TX and GX layers consistently provide strong signals for C/N classification and early‐stage detection, JX data capture critical metabolic alterations, performing comparably to TX for C/N classification. Markers such as THBS3 and SEMA5A, reflecting shifts toward enhanced glycolysis and altered extracellular matrix interactions, further validate the central role of metabolic reprogramming in lung cancer. Additionally, distinct marker signatures emerge across cancer subtypes; for instance, differences in the expression of ITGB5, CLDN11, and KRT5 between AD and SC highlight subtype‐specific metabolic adaptations affecting cell adhesion and signaling, while noncoding RNAs (e.g., LOC100130744, LINC01020) suggest further layers of post‐transcriptional regulation that influence tumor behavior.

Our efforts to enhance the efficacy of JX data for distinguishing lung cancer subtypes and stages employed a series of ML experiments that combined various sampling techniques, data types, classification algorithms, and normalization methods. The performance metrics, as summarized in Table 1 and illustrated by ROC curves in Figures S5 and S6 in Supporting Information S1, revealed marked disparities among datasets. The TCGA dataset exhibited exceptionally high performance (> 95% accuracy across metrics), emphasizing its reliability. In contrast, the CCLE data showed poor separation, reflecting limitations that likely arise from differences in experimental conditions, data quality, or inherent platform characteristics. Similarly, while the NCBI‐GEO dataset generally achieved high precision and recall, outlier values in certain subtype (SC) classifications—such as a recall of 0.14—indicate challenges in consistently capturing all positive instances. Additionally, F1‐scores and recall metrics showed higher variability in both NCBI‐GEO and CCLE datasets, largely due to class imbalance [100]. This problem arose from unbalanced distribution of data across subgroups and all data sets we used contained more or less represented subgroups. Despite applying techniques such as oversampling, balanced random sampling, and cost‐sensitive learning, the variability in classification metrics persisted [53]. These findings highlight the need for further improvements in model tuning or selection, along with model‐based experimental designs—to enhance predictive performance and reliability.

Feature importance analysis using SHAP values (Figure 6, Table 2) consistently highlighted these established and novel markers, confirming that the framework not only validates known metabolic alterations—such as those underlying the Warburg effect—but also uncovers novel candidates for clinical exploration. In C/N analysis, a hierarchy of alterations begins with mitochondrial amino acid metabolism and progresses to lipid oxidation and phospholipid biosynthesis. For SCLC/NSCLC classifications, emerging markers such as APAT2rm suggest rewiring in amino acid metabolism linked to β‐alanine pathways and treatment resistance, while ABTArm’s immune‐related role presents opportunities for improving immunotherapy outcomes. In AD/SC classifications, novel markers, like steryl‐sulfatase (RE3220C), offer insights into hormonal influences, and components of nucleotide metabolism further distinguish AD’s proliferative demands from SC. Overall, this comprehensive approach refines our understanding of lung cancer metabolism, paving the way for targeted therapeutic interventions, improved diagnostic precision, and personalized treatment strategies.

For PC, despite the reduced sample size, the feature distributions across omic layers (TX, JX, and PX) remained consistent with those observed in lung cancer, and integrated classifier consistently achieved high accuracy, with TX and JX models often matching or exceeding performance metrics of individual layers. The functional enrichment analysis of features with highest absolute mean SHAP values revealed several novel candidate biomarkers that refine the disease’s metabolic signature (Figure 8). Together, these novel markers complement established oncogenic processes and offer promising avenues for improved early detection and targeted therapies. This refined signature underscores the utility of our multi‐omic pipeline in uncovering clinically relevant biomarkers and demonstrates its potential to inform translational research and personalized medicine in PC.

In conclusion, the proposed multi‐omic integration and classification method effectively identified key metabolic pathways and achieved high classification accuracy across different cancer subtypes and early‐stage cancer. By leveraging the strengths of GSMMs and integrating information from various omic data layers, this methodology provides a comprehensive understanding of cancer metabolism and highlights critical pathways which could point to potential therapeutic targets. This approach underscores the importance of multi‐omic data integration in improving cancer diagnosis, subtyping, and early staging. Given the extensive research conducted at different omic levels in lung cancer, a large number of samples are available, and much is known about its mechanisms. Consequently, lung cancer was chosen to measure the performance of the methodology using datasets with abundant sample sizes. After achieving success with lung cancer, PC was selected to test the methodology’s performance in a group with fewer samples. Despite its lower prevalence, PC is fundamentally similar in terms of metabolic or other omic levels. This allowed for the assessment of the methodology in a disease with fewer samples, demonstrating its applicability to less well‐known diseases.

## 4 MATERIALS AND METHODS

### 4.1 Data acquisition and preprocessing

Lung cancer gene expression data published in the TCGA database in next‐generation sequencing format (RNA‐seq) from different projects (TCGA‐LUAD, TCGA‐lung squamous cell carcinoma (LUSC) surveillance, epidemiology, and end results (SEER) program. Cancer stat facts: lung and bronchus cancer, “National Cancer Institute Cancer Model Development for the Human Cancer Model Initiative—HCMI‐CMDC,” and “National Cancer Institute Clinical Proteomic Tumor Analysis Consortium—CPTAC‐3”) were compiled (Figure S1A). The TCGA dataset contains a total of 305 healthy (N) tissue samples and 1269 tumor tissue (C) samples. Among the 656 AD samples, 359 were classified as stage I (S1), 155 as stage II (S2), 106 as stage III (S3), and 28 as stage IV (S4). Among the 613 squamous cell neoplasm (SC) samples, 284 were classified as S1, 206 as S2, 105 as S3, and 10 as S4. For the remaining samples, staging information is not available in the database and thus were not included in the staging classifications. In addition, TX data published for 86 samples (7 normal, 79 SCLC) from ArrayExpress [101] were included (accession ID E‐GEOD‐60052) [102]. RNAseq data in FPKM‐uq (fragments per kilo base of transcript per million mapped fragments‐upper quartile) format was used. The expression data were normalized with respect to the PKM gene.

This dataset was later augmented with microarray expression data from tissue samples from the “National Center for Biotechnology Information—Gene Expression Omnibus” (NCBI‐GEO) [47] database and cell line samples from the “Cancer Cell Line Encyclopedia” (CCLE) database (Figure S1C,D) [50]. All datasets were selected to belong exclusively to the Affymetrix platform to ensure absolute signal ranges and annotation consistency. Due to limited staging information in the NCBI‐GEO data, staging was omitted from the microarray dataset. The final GEO dataset includes 93 normal (N) samples, 764 AD samples, 221 SC samples, 53 LaC samples, and 41 SCLC samples (Table S1). In addition to the datasets created with primary tissue samples, a total of 87 lung cancer cell line microarray data were downloaded from the CCLE database and grouped according to their subtypes to create a model for cell lines. Samples without gene expression data were excluded from the workflow, resulting in 87 out of 107 samples matching the study design being selected. These 87 samples are distributed as follows: 45 AD, 7 LaC, 16 SC, 13 SCLC, and 6 N.

For the application of PC and classification using additional omic layers, available transcriptomic (TX: RNAseq), genomic (GX: CNV), proteomic (PX: RPPA) and clinical data of 1139 samples of lung cancer (TCGA‐LUAD, TCGA‐LUSC) and 161 (TCGA‐PAAD‐CPTAC‐3) samples of PC were used.

GX data as gene‐level CNV files were downloaded and processed with GISTIC2 [103]. The dataset comprises 501 SC samples, 516 AD samples, and 184 PC samples, with each sample containing information on approximately 25,000 genes. At the Genomic Data Commons database, PX data was published as RPPA .tsv files [104]. RPPA data for AD, SC, and PC included 223, 223, and 124 samples, respectively, each containing 365, 328, and 195 proteins.

### 4.2 Human Recon3D and integration of transcriptome data

Collected TX data were integrated into the Recon3D human GSMM [28, 42] using the E‐flux method [35] to obtain dataset‐specific flux distributions. The flux bounds were set based on a priori determined GPR rules. The “minSum” method was employed for this purpose, utilizing the minimum value of gene expression for genes related to the “AND” rule (enzyme complexes) and the sum of expression values for genes related to the “OR” rule (isozymes) ($lb_{j}$ and $ub_{j}$ for reaction j with defined expression value, in Equation 1). FBA was performed to obtain flux distributions (v in Equation 1) by maximizing growth ($c^{T}v$), which can be used as objective for cancer cells because their main aim is proliferate and invade other tissues [105, 106].

(1) $$\begin{array}{cc} \max & c^{T}v\\ \text{subject}\;\text{to} & \left\{\begin{array}{c} S\cdot v=0\\ lb_{j}\leq v_{j}\leq ub_{j} \end{array}\right. \end{array},$$

where S is the stoichiometric matrix. Since the samples were extracted from tissues, a semi‐quantitative approach was used when constraining the model, allowing the uptake of amino acids, growth factors such as biotin and choline, glucose, and oxygen. The growth reaction was maximized to obtain flux distributions with the given constraints for all individual samples, using cobrapy [107] and equipped with Gurobi solver. Additionally, various objective functions, such as biomass maintenance and lactate production, both known to be influenced by the Warburg effect [30] were explored to optimize the dataset for maximum classification performance and biological relevance. The resulting outputs were analyzed at the pathway level, incorporating the expression values of genes encoding the protein complexes that catalyze each reaction (referred to as reaction gene expression values). This analysis was aligned with the “hallmarks of cancer” to ensure a comprehensive and meaningful interpretation of metabolic changes.

### 4.3 Differential and functional analyses of fluxomic data

The datasets were grouped based on subtypes (N: normal, C: cancer, SCLC, NSCLC, AD, SC) and stages (Stages I (S1), II (S2), III (S3) and IV (S4)):

1. For lung cancer: 36 groups consisting C‐N, SCLC‐NSCLC, 3 subtypes (N‐AD‐SC), AD‐N, SC‐N, SC‐AD, AD‐all stages, SC‐all stages, for AD and SC stage 2‐1, 3‐1, 4‐1, 3‐2, 4‐2, 4‐3 and for all stages individually—AD‐N, SC‐N, AD‐SC.
2. For PC: two groups consisting N‐C, N‐S1.

All of the cutoff values were determined considering the distribution of related metrics. Initially, the distribution of the JX data was checked using Lilliefors test [108], and based on the findings showing that data is not normally distributed (*p* < 1e‐5), the statistical significance of differences between binary groups was assessed using the Wilcoxon rank sum test (Mann–Whitney *U* test) for two independent samples, and the Kruskal–Wallis test for the analysis of multiple groups (N‐AD‐SC and intra‐subspecies stages) [109, 110, 111]. All resulting *p*‐values from the analyses and the chosen alpha value of 0.05 were adjusted using the Benjamini–Hochberg method (FDR) to reduce type 1 errors/false positives in multiple comparisons [112]. The same procedures were repeated for the gene expression values calculated for each reaction to determine if the results were consistent with the changes in gene expression provided as constraints to the model.

Pathway enrichment analysis for reactions was conducted in MATLAB using the hypergeometric test [113]. The hypergeometric test can be used to determine which types of samples are overrepresented in a subgroup of n samples selected from a population. Here, the subgroup is the list of reactions with statistically significant expression values or fluxes, and the feature refers to the pathways (subsystems) defined in the model for these reactions. The formula used for the hypergeometric test is expressed as Equation (2).

(2) $$P\left(X\right)=\frac{\left(\begin{array}{c} m\\ x \end{array}\right)\left(\begin{array}{c} N-m\\ n-x \end{array}\right)}{\left(\begin{array}{c} N\\ m \end{array}\right)}.$$

In the formula, N represents the total number of reactions, n represents the number of statistically significant reactions, m represents the total number of reactions in a pathway, and x represents the number of reactions associated with that pathway among the significant reactions. Pathways that are significantly overrepresented (*p* < 0.1) are shown in the heatmap. The results obtained were qualitatively compared with the known characteristics of cancer (“hallmarks of cancer”) in the literature [92, 99].

### 4.4 Development of single‐omic and multi‐omic classifiers

During investigation of the potential of JX data in classification of cancer, various ML models were constructed such as random forests (RFs) and support vector machines (SVMs) [52, 53]. Different ML models were compared to achieve the best classification performance in both subtyping (AD vs. SC vs. N) and staging. The comparison was based on different performance criteria (accuracy, precision, recall, F1‐score, AUC, etc.) on test data that is not included in the model’s training. These models were used for the same sequential binary and/or multi‐group classifications that are used in statistical analyses. The models were implemented using the Python programming language and the Scikit‐learn library [114]. All models underwent hyperparameter optimization to prevent overfitting using GridSearchCV algorithm (n_estimators: the number of trees, max_depth: the maximum depth of the tree, max_features: the number of features to consider when looking for the best split, min_samples_leaf: the minimum number of samples required to be at a leaf node, min_samples_split: the minimum number of samples required to split an internal node) [54]. 80% of the samples in the dataset were used as training data for model construction, while 20% were held out as test data to measure model performance. Stratified cross‐validation was used during the setup of RF and SVM models.

During the enhancement of the method, a voting classifier was established to assess the impact of each omic level on prediction success using the combined omic data from PC and LC compiled datasets. This classifier aggregates the predictions from each sub‐classifier (RF models of trained using each omic data) and calculates the metrics to analyze their effects.

In voting classifier method, $X_{1}$, $X_{2},\text{…},X_{m}$ represents m omics data matrices (e.g., GX, TX, JX, and PX) for N samples, where $X_{m}\in R^{N\times d_{m}}$ and $d_{m}$ is the number of features in the mth omics layer. A distinct random forest classifier $f_{m}$ was trained on each $X_{m}$ using scaled data without feature selection to estimate class probabilities (Equation 3).

(3) $$P_{m}\left(y=k|X_{m}\right)=\frac{1}{T}\;\sum_{t=1}^{T}I\left(h_{m,t}\left(X_{m}\right)=k\right),$$

where $h_{m,t}$ is the tth tree in the mth RF and k∈{1,…,K} denotes class labels. The final prediction combines probabilities across omics layers via weighted averaging (Equation 4).

(4) $$\bar{P}\left(y=k|X_{1},\ldots ,X_{m}\right)=\sum_{m=1}^{M}w_{m}P_{m}\left(y=k|X_{m}\right),\sum_{m=1}^{M}w_{m}=1,$$

Weights $w_{m}$ can be uniform or optimized via grid search, however, in this case weights were uniform.

SHAP (SHapley Additive exPlanations) values were utilized to identify reactions, genes, and proteins of high importance (mean|SHAP| > 1e‐4) in the classification of each subgroup, which could potentially serve as biomarkers across all results [115]. Following the identification of important features, a functional evaluation of the selected genes and proteins was conducted through Gene Ontology (GO) analysis using the clusterProfiler package in the R programming language [116, 117]. This approach facilitated the identification of GO parameters linked to biological processes associated with features exhibiting statistically significant changes in their levels.

## AUTHOR CONTRIBUTIONS

**Ezgi Tanıl**: Conceptualization; data curation; formal analysis; investigation; methodology; validation; visualization; writing—original draft. **Emrah Nikerel**: Conceptualization; formal analysis; funding acquisition; investigation; project administration; resources; supervision; writing—review and editing.

## CONFLICT OF INTEREST STATEMENT

The authors declare no conflicts of interest.

## ETHICS STATEMENT

This study does not include any methodology that requires animal or human materials.

## Supporting information

Supporting Information S1

## Data Availability

The datasets utilized in this study are publicly available from the following repositories: TCGA (portal.gdc.cancer.gov), NCBI‐GEO (ncbi.nlm.nih.gov/geo), ArrayExpress (ebi.ac.uk/biostudies/arrayexpress), and CCLE (sites.broadinstitute.org/ccle/). The codes employed for the analyses can be accessed at GitHub website (nikerellab/ModimSEDC).
