# Supplementary material for: Multi‐omic data integration and exploiting metabolic models using systems biology approach increase precision in subtyping and early diagnosis of cancer
Source: Quant Biol. 2025 Aug 5;13(4):e70012. doi: 10.1002/qub2.70012 (PMC12806132; doi:10.1002/qub2.70012)
Supplement: Supplementary file 1 — Supporting Information S1 [file QUB2-13-e70012-s001.pdf]

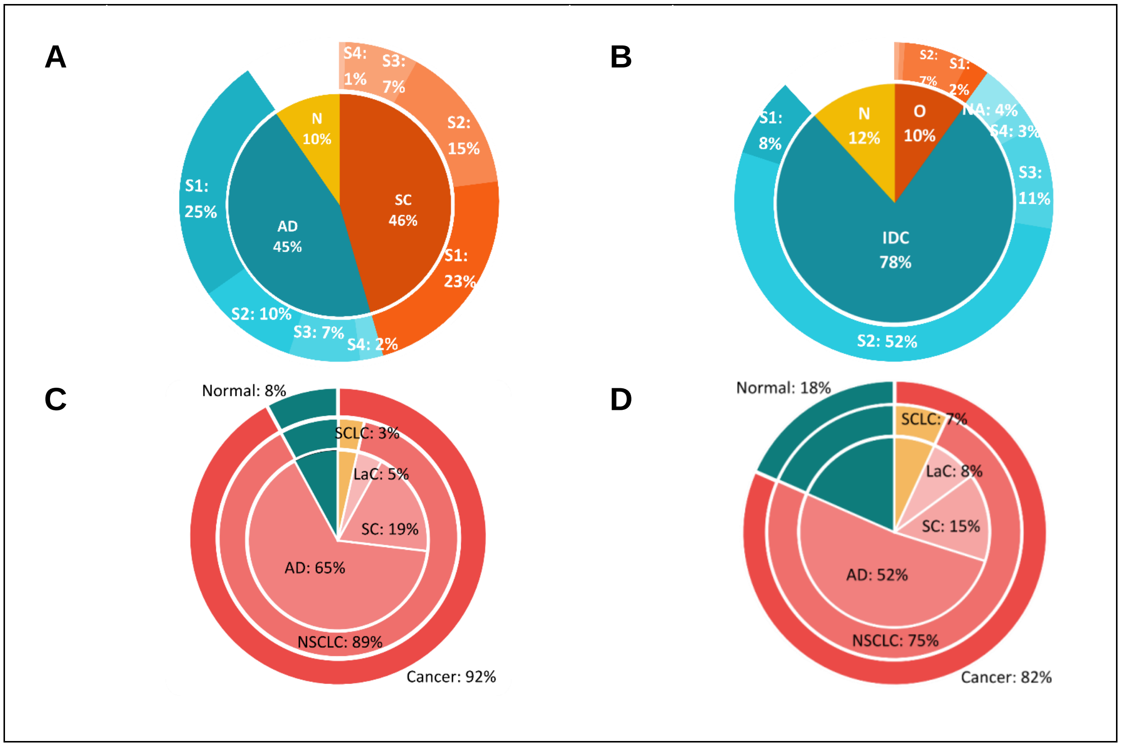

Figure S1. Distribution of cancer types, stages, and conditions across: (A) Lung cancer RNA-seq patient tissue samples from TCGA (N: Normal, C: Cancer, AD: Adenomas and Adenocarcinomas SC: Squamous cell neoplasms, S1,S2,S3,S4: Stage i,ii,iii,iv) (B) Pancreatic cancer RNA-seq patient tissue samples from TCGA (IDC: Invasive Ductal Carcinoma, O: Other) (C) Lung cancer microarray patient tissue samples from NCBI-GEO (SCLC: Small Cell Lung Cancer, NSCLC: Non-Small Cell Lung Cancer, LaC: Large Cell Lung Cancer) (D) CCLE microarray lung cancer cell culture samples

Table S1. Accession numbers and references of GEO lung cancer gene expression samples

GEO ID	N	C	AD	SC	LaC	SCLC	Ref
GSE10245	-	58	40	18	-	-	[1]
GSE10445	-	72	72	-	-	-	[2]
GSE10799	3	16	16	-	-	-	[3]
GSE118370	6	6	6	-	-	-	[4]
GSE12667	-	75	68	1	4	-	[5]
GSE149507	18	18	-	-	-	18	[6]
GSE18842	-	91	14	31	-	-	[7]
GSE19188	65	91	45	27	19	-	[8]
GSE19804	-	120	Not Defined				[9]
GSE28571	-	100	50	28	22	-	[10]
GSE31210	-	246	246	-	-	-	[11]
GSE43346	1	23	-	-	-	23	[12]
GSE43580	-	150	77	73	-	-	[13]
GSE50081	-	181	130	43	8	-	[14]

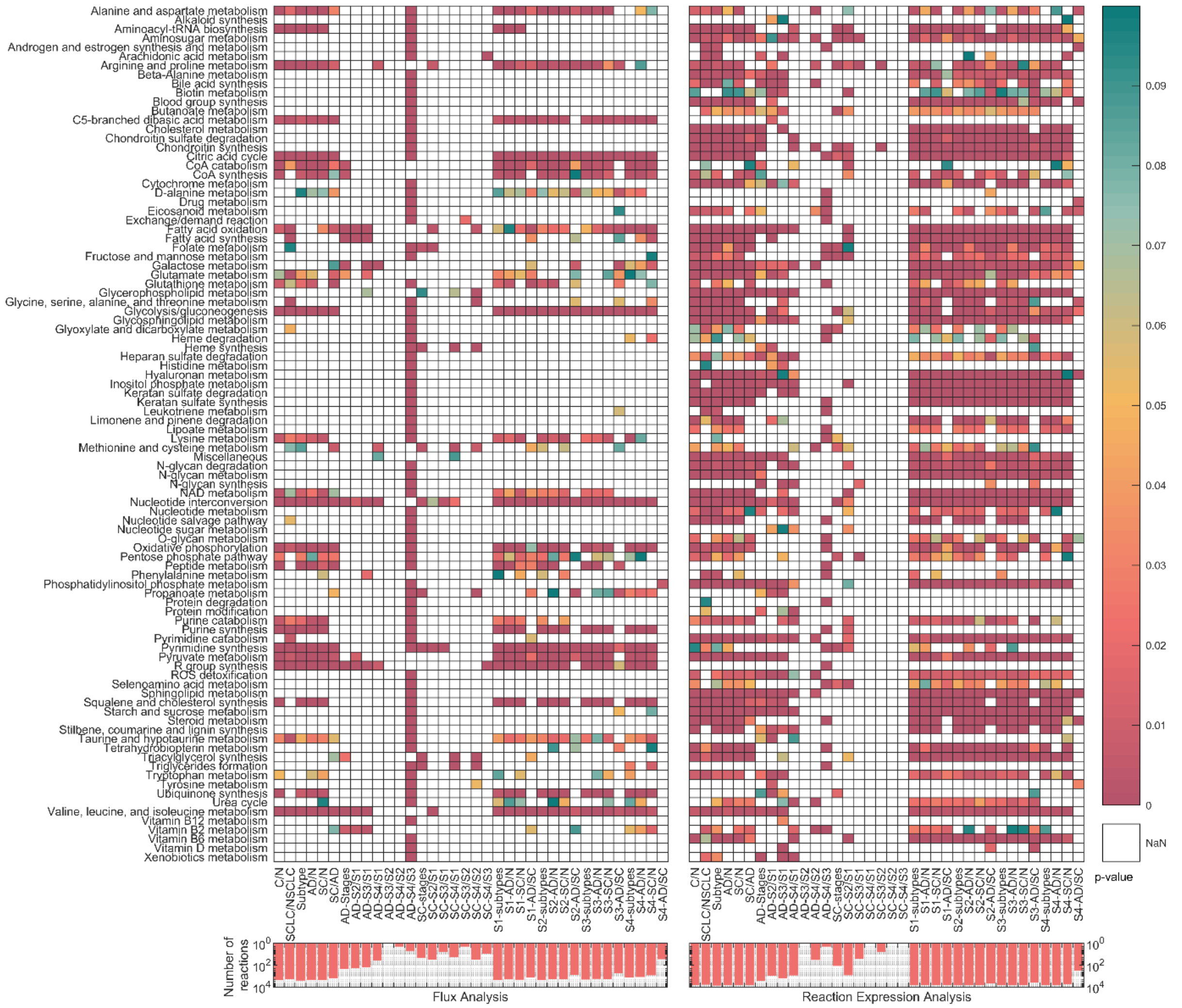

Figure S2. Differential flux and pathway enrichment analysis results of the TCGA dataset, including staging groups. Over-represented subsystem terms for classification groups are displayed in heatmaps, and reactions with significantly different flux and reaction expression values are illustrated in bar charts.

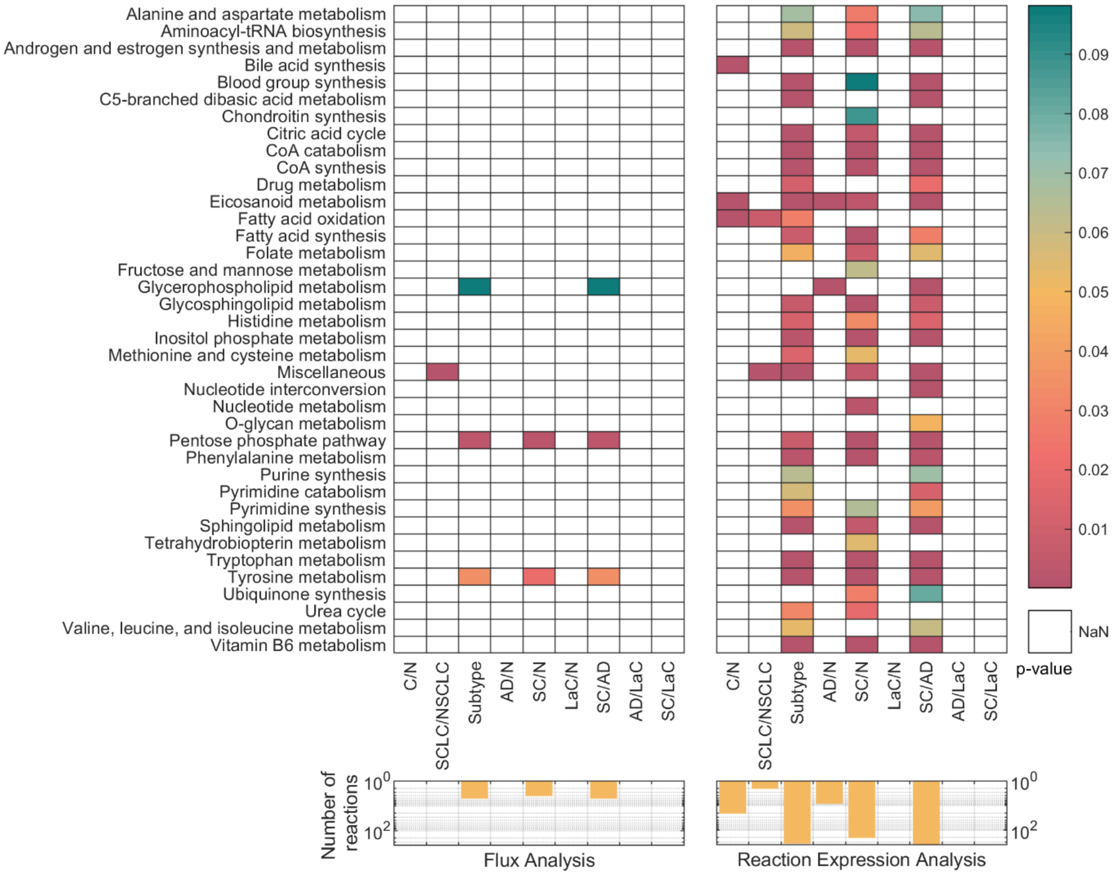

Figure S3. Differential flux and pathway enrichment analysis results of the CCLE dataset. Over-represented subsystem terms for classification groups are displayed in heatmaps, and reactions with significantly different flux and reaction expression values are illustrated in bar charts.

Table S2. Key reactions with highest SHAP values for subgroup classification using fluxome data across different omic layers for lung cancer

			Abbreviation	Name	Subsystem/Process
LC	C/N	GX	*THBS3*	Thrombospondin 3	Cell-matrix adhesion
			SEMA5A	Semaphorin 5A	Cell adhesion / blood vessel endothelial cell proliferation involved in sprouting angiogenesis
			GBAP1	Glucosylceramidase Beta 1 Like, Pseudogene	Sphingolipid metabolism/autophagy
			PBRM1	Polybromo 1	Mitotic cell cycle/chromatin remodeling/T-cell differentiation
			ELAVL2	ELAV Like RNA Binding Protein 2	Regulation of DNA-templated transcription
		TX	*STX11*	Syntaxin 11	Protein transport
			SAPCD2	Suppressor APC Domain Containing 2	Cell division
			FAM107A	Family With Sequence Similarity 107 Member A	Cell growth/migration
			GPR146	G Protein-Coupled Receptor 146	G protein-coupled receptor signaling pathway/regulation of cholesterol biosynthetic process
			CDCA3	Cell Division Cycle Associated 3	Cell division/protein ubiquitination
		JX	ESTRONESt	Transport of Estrone 3-Sulfate via Bicarbonate Antiport	Transport, extracellular
			G3PD1	Glycerol-3-Phosphate Dehydrogenase (NAD)	Glycerophospholipid metabolism
			SERPT	Serine C-Palmitoyltransferase	Sphingolipid metabolism
			FACOAL1821	Fatty-Acid- Coenzyme A Ligase	Fatty acid oxidation
			r1393	Glycogen Phosphorylase	Starch and sucrose metabolism
	S1/N	GX	*LOC100130744*	non-coding RNA	-
			LINC01020	Long Intergenic Non-Protein Coding RNA 1020	Associated with Hereditary Hemorrhagic Telangiectasia
			S100A7L2	S100 Calcium Binding Protein A7 Like 2	Endothelial cell migration
			MIR887	MicroRNA 887	miRNA-mediated post-transcriptional gene silencing
			FAM134B	Reticulophagy Regulator 1	autophagy/ER organization
		TX	*GPT2*	Glutamic--Pyruvic Transaminase 2	L-alanine metabolic process
			PTPN21	Protein Tyrosine Phosphatase Non-Receptor Type 21	Chromatin remodeling
			GLIPR2	GLI Pathogenesis Related 2	positive regulation of epithelial cell migration
			GRK5	G Protein-Coupled Receptor Kinase 5	G protein-coupled receptor signaling pathway/apoptotic process
			INMT	N-Methyltransferase	Amine metabolic process/methylation
		JX	r0202	Sn-Glycerol-3-Phosphate:NAD+ 2-Oxidoreductase	Glycolysis/gluconeogenesis
			GLBRAN	1, 4-Alpha-Glucan Branching Enzyme (Glygn1 -> Glygn2)	Starch and sucrose metabolism

Table S2. (Continued)

			Abbreviation	Name	Subsystem/Process
LC	SC/AD	GX	*ITGB5*	Integrin Subunit Beta 5	integrin-mediated signaling pathway/cell adhesion
			CLDN11	Claudin 11	Cell adhesion
			GNB4	G Protein Subunit Beta 4	G protein-coupled receptor signaling pathway
			PARP14	Poly(ADP-Ribose) Polymerase Family Member 14	Regulation of tyrosine phosphorylation of STAT protein
			CPOX	Coproporphyrinogen Oxidase	Porphyrin-containing compound metabolism
		TX	*PVRL1*	Nectin Cell Adhesion Molecule 1	Cell junction organization/cell adhesion
			CERS3	Ceramide Synthase 3	Sphingolipid metabolic process
			DSG3	Desmoglein 3	Apoptotic cleavage of cellular proteins
			KRT5	Keratin 5	Cell junction organization
			BNC1	Basonuclin Zinc Finger Protein 1	Regulation of transcription by RNA polymerase I
		JX	r2132	Major Facilitator (Mfs) Tcdb:2.A.1.13.1	Transport, extracellular
			BTNt2	Biotin Reversible Transport via Proton Symport	Transport, extracellular
			HMR_3219	Enoyl Coenzyme A Hydratase	Fatty acid oxidation
			FACOAL1831	Fatty-Acid- Coenzyme A Ligase	Fatty acid oxidation
		PX	MIG-6	Mitogen-Inducible Gene 6 Protein	protein kinase binding and kinase binding.
			IGFBP2	Insulin Like Growth Factor Binding Protein 2	insulin-like growth factor binding
			Napsin-A	Napsin A Aspartic Peptidase	aspartic-type endopeptidase activity
			PCNA	Proliferating Cell Nuclear Antigen	DNA repair
			AMPK, Alpha	Protein Kinase AMP-Activated Catalytic Subunit Alpha 1	response to hypoxia

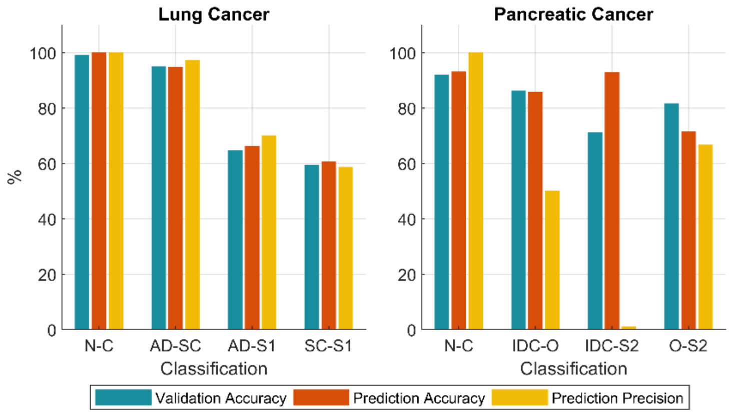

Figure S4. The performance metrics achieved for lung and pancreatic cancer subtype classifications using SVM models. The classifications for lung cancer include N-C, AD-SC, AD-S1, and SC-S1, while pancreatic cancer classifications consist of N-C, IDC-O , IDC-S2, and O-S2. The y-axis represents the percentage (%) of model performance, and the x-axis denotes classification categories.

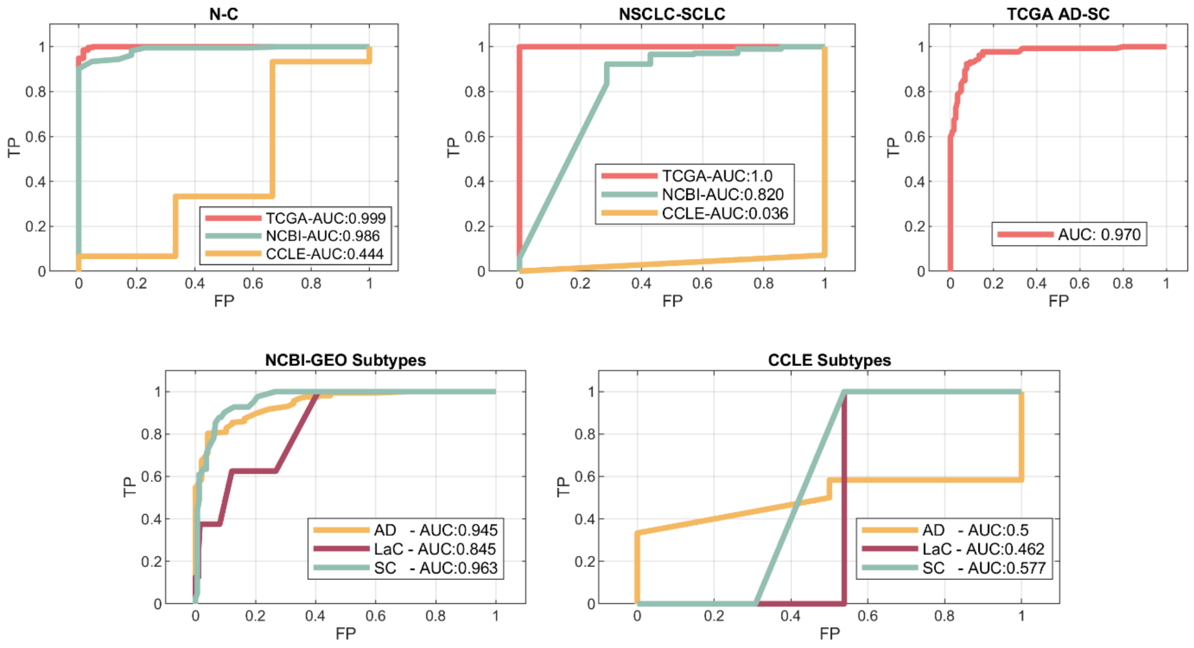

Figure S5. Receiver Operating Characteristic (ROC) curves of fluxome classifiers for various datasets and classification tasks. The curves plot the True Positive Rate (TP, sensitivity) against the False Positive Rate (FP-specificity), illustrating the trade-off between sensitivity and specificity for each model.

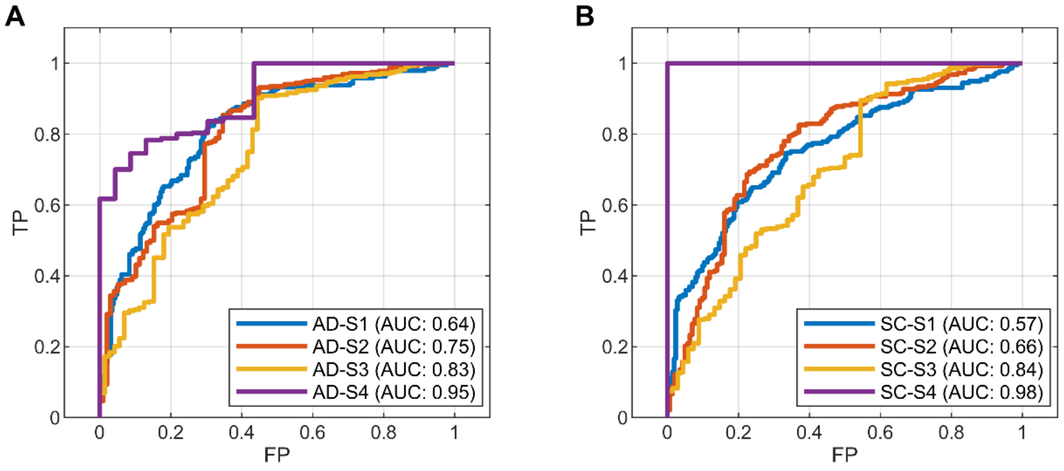

Figure S6: ROC Curves for classification of (A) AD stages (B) SC stages. The curves plot the True Positive Rate (TP, sensitivity) against the False Positive Rate (FP, specificity), illustrating the trade-off between sensitivity and specificity for each model.

Table S3. Key reactions with highest SHAP values for subgroup classification using fluxome data across different omic layers for pancreatic cancer

			Abbreviation	Name	Subsystem/Process
PC	C/N	TX	*CDC25C*	Cell Division Cycle 25C	G2/M transition of mitotic cell cycle
			RACGAP1	Rac GTPase Activating Protein 1	Mitotic cytokinesis/signal transduction
			SORCS1	Sortilin Related VPS10 Domain Containing Receptor 1	post-Golgi vesicle-mediated transport/neuropeptide signaling
			LMO7	LIM Domain 7	protein ubiquitination
			TBC1D2	TBC1 Domain Family Member 2	positive regulation of GTPase activity
		JX	MCDm	Malonyl Coenzyme A Decarboxylase, Mitochondrial	Fatty acid oxidation
			MANt4	D-Mannose Transport via Sodium Symport	Transport, extracellular
			G6PDH2r	Glucose 6-Phosphate Dehydrogenase	Pentose phosphate pathway
		PX	AGPS	Alkylglycerone Phosphate Synthase	lipid metabolic process
			OSBPL3	Oxysterol Binding Protein Like 3	lipid transport
			HK2	Hexokinase 2	carbohydrate metabolic process
			GNPAT	Glyceronephosphate O-Acyltransferase	fatty acid metabolic process
			HSCB	HscB Mitochondrial Iron-Sulfur Cluster Cochaperone	iron-sulfur cluster assembly
			LAMC2	Laminin Subunit Gamma 2	cell adhesion
			BAIAP2L1	BAR/IMD Domain Containing Adaptor Protein 2 Like 1	plasma membrane organization
			MYEF2	Myelin Expression Factor 2	neuron differentiation
			BCAM	Basal Cell Adhesion Molecule (Lutheran Blood Group)	angiogenesis/cell adhesion
			MICALL2	MICAL Like 2	actin cytoskeleton organization
	S1/N	TX	*ACSL4*	Acyl-CoA Synthetase Long Chain Family Member 4	long-chain fatty acid metabolic process
			CEP68	Centrosomal Protein 68	centrosome cycle
			EPHX2	Epoxide Hydrolase 2	lipid metabolic process
			NT5DC4	5'-Nucleotidase Domain Containing 4	
			CTNNA1	Catenin Alpha 1	cell adhesion
		JX	RE3597C	Carbonyl Reductase (NADPH)	Eicosanoid metabolism
			r0464	4-Aminobutyraldehyde:NAD+ Oxidoreductase	Glutamate metabolism
			r0475	2-Deoxyadenosine 5-Diphosphate:Oxidized-Thioredoxin 2-Oxidoreductase	Nucleotide interconversion
			SERGLNexR	L-Serine/Glutamine Reversible Antiport	Transport, extracellular
			MCCCrm	Methylcrotonoyl Coenzyme A Carboxylase, Mitochondrial	Valine, leucine, and isoleucine metabolism

References

1. Kuner R, Muley T, Meister M, et al (2009) Global gene expression analysis reveals specific patterns of cell junctions in non-small cell lung cancer subtypes. Lung Cancer 63:32–38. https://doi.org/10.1016/j.lungcan.2008.03.033
2. Broët P, Camilleri-Broët S, Zhang S, et al (2009) Prediction of Clinical Outcome in Multiple Lung Cancer Cohorts by Integrative Genomics: Implications for Chemotherapy Selection. Cancer Res 69:1055–1062. https://doi.org/10.1158/0008-5472.CAN-08-1116
3. Wrage M, Ruosaari S, Eijk PP, et al (2009) Genomic Profiles Associated with Early Micrometastasis in Lung Cancer: Relevance of 4q Deletion. Clin Cancer Res 15:1566–1574. https://doi.org/10.1158/1078-0432.CCR-08-2188
4. Xu L, Lu C, Huang Y, Zhou J, Wang X, Liu C, Chen J, Le H (2018) SPINK1 promotes cell growth and metastasis of lung adenocarcinoma and acts as a novel prognostic biomarker. BMB Rep 51:648–653. https://doi.org/10.5483/BMBRep.2018.51.12.205
5. Ding L, Getz G, Wheeler DA, et al (2008) Somatic mutations affect key pathways in lung adenocarcinoma. Nature 455:1069–1075. https://doi.org/10.1038/nature07423
6. Cai L, Liu H, Huang F, et al (2021) Cell-autonomous immune gene expression is repressed in pulmonary neuroendocrine cells and small cell lung cancer. Commun Biol 4:314. https://doi.org/10.1038/s42003-021-01842-7
7. Sanchez-Palencia A, Gomez-Morales M, Gomez-Capilla JA, Pedraza V, Boyero L, Rosell R, Fárez-Vidal ME (2011) Gene expression profiling reveals novel biomarkers in nonsmall cell lung cancer. Int J Cancer 129:355–364. https://doi.org/10.1002/ijc.25704
8. Hou J, Aerts J, den Hamer B, et al (2010) Gene Expression-Based Classification of Non-Small Cell Lung Carcinomas and Survival Prediction. PLoS One 5:e10312. https://doi.org/10.1371/journal.pone.0010312
9. Lu T-P, Hsiao CK, Lai L-C, Tsai M-H, Hsu C-P, Lee J-M, Chuang EY (2015) Identification of regulatory SNPs associated with genetic modifications in lung adenocarcinoma. BMC Res Notes 8:92. https://doi.org/10.1186/s13104-015-1053-8
10. Jabs V, Edlund K, König H, et al (2017) Integrative analysis of genome-wide gene copy number changes and gene expression in non-small cell lung cancer. PLoS One 12:e0187246. https://doi.org/10.1371/journal.pone.0187246
11. Yamauchi M, Yamaguchi R, Nakata A, et al (2012) Epidermal Growth Factor Receptor Tyrosine Kinase Defines Critical Prognostic Genes of Stage I Lung Adenocarcinoma. PLoS One 7:e43923. https://doi.org/10.1371/journal.pone.0043923
12. Sato T, Kaneda A, Tsuji S, et al (2013) PRC2 overexpression and PRC2-target gene repression relating to poorer prognosis in small cell lung cancer. Sci Rep 3:1911. https://doi.org/10.1038/srep01911
13. Tarca AL, Lauria M, Unger M, et al (2013) Strengths and limitations of microarray-based phenotype prediction: lessons learned from the IMPROVER Diagnostic Signature Challenge. Bioinformatics 29:2892–2899. https://doi.org/10.1093/bioinformatics/btt492
14. Der SD, Sykes J, Pintilie M, Zhu C-Q, Strumpf D, Liu N, Jurisica I, Shepherd FA, Tsao M-S (2014) Validation of a Histology-Independent Prognostic Gene Signature for Early-Stage, Non–Small-Cell Lung Cancer Including Stage IA Patients. J Thorac Oncol 9:59–64. https://doi.org/10.1097/JTO.0000000000000042
